# Mapping the oxidative landscape in cystic fibrosis: methodological frontiers and application

**DOI:** 10.3389/fphar.2025.1632924

**Published:** 2025-07-16

**Authors:** Michela Rubin, Ilaria Artusi, Giorgio Cozza

**Affiliations:** ^1^ Department of Molecular Medicine (DMM), University of Padua, Padua, Italy; ^2^ Department of Pharmaceuticals and Pharmacological Sciences (DSF), University of Padua, Padua, Italy; ^3^ Biostructures and Biosystems National Institute (INBB), Rome, Italy

**Keywords:** cystic fibrosis, oxidative stress, inflammation, analytic techniques, ROS

## Abstract

Cystic Fibrosis (CF), a multi-organ disease stemming from CFTR gene mutations, is characterized by progressive pulmonary disease, chronic inflammation, and a pro-oxidative environment. The intricate relationship between CFTR dysfunction, oxidative stress, and inflammation underscores the need to accurately characterize oxidative stress markers to identify therapeutic targets. This review compiles and analyzes methodologies employed in the CF field for this purpose, presenting selected applications and outcomes while highlighting potential inconsistencies due to experimental variations. The review encompasses a wide array of analytical techniques. These include methods for direct reactive oxygen species (ROS) detection (e.g., superoxide, hydrogen peroxide), characterization of oxidative damage to lipids (e.g., TBARS, F2-isoprostanes; lipidomics), proteins (e.g., carbonylation, S-nitrosylation, S-glutathionylation; proteomics), and DNA (e.g., 8-OHdG). Assays for major non-enzymatic antioxidants (glutathione, vitamins), enzymatic antioxidant systems (superoxide dismutase, catalase, glutathione peroxidase), and total antioxidant capacity (TAC) are detailed. Furthermore, methods to assess mitochondrial function for studying oxidative stress in CF are discussed. The critical choice of experimental models (*in vitro*, *in vivo*) and biological samples (e.g., blood, sputum, BALF, EBC, cells), along with their specific considerations, are also integral to the review. Application of these diverse methodologies frequently reveals heightened oxidative stress and perturbed antioxidant defenses across various CF-relevant compartments, although results can be influenced by the specific model or technique utilized. Ultimately, this comprehensive analysis underscores the complexity of assessing oxidative stress in CF and strongly advocates for the implementation of integrated, multiparametric strategies. Such synergistic approaches, combining complementary methodologies, are crucial for a holistic understanding of redox dysregulation, facilitating the identification of reliable biomarkers, and guiding the development of more effective, targeted antioxidant therapies to improve clinical outcomes in CF.

## 1 Introduction

Cystic fibrosis (CF), caused by mutations in the cystic fibrosis conductance regulator (*CFTR*) gene, is the most common rare disease affecting the Caucasian population, with an incidence of 2,500–3,500 newborns (Bobadilla et al., 2002). Functional CFTR is located on the apical membrane of epithelial cells, where it conducts chloride and bicarbonate ions towards the extracellular space. Its activity helps in regulating water content on body surfaces (Saint-Criq and Gray, 2017). Impairment in CFTR functionality is associated to loss of tissue homeostasis that leads to the accumulation of viscous mucus creating the ideal environment for bacterial infections and inflammation. Entrapped pathogens and particles stimulate a robust immune response, prompting cells like neutrophils and macrophages to secrete pro-inflammatory cytokines for host defense. Moreover, in CF, airway epithelial cells are characterized by an altered chemokines production, that consistently contributes to neutrophils attraction and activation (Ghigo et al., 2021). Despite the intense inflammatory response generated, CF patients are paradoxically unable to overcome the infection (Bals et al., 1999). Neutrophils, while abundant, can become dysfunctional within the unique, hypoxic, and nutrient-poor mucus environment. This leads to a state of frustrated phagocytosis and the release of damaging proteases and reactive oxygen species (ROS) that cause significant collateral damage to the host tissue without effectively clearing the bacteria, which are often protected within biofilms (Gifford and Chalmers, 2014). Consequently, instead of resolving the infection, these persistent pro-inflammatory events perpetuate a vicious cycle, pushing the local environment towards a chronic and damaging pro-oxidative state. These conditions contribute to tissue degeneration and organ failure, thus making CF a multi-organ disease. Anyway, progressive pulmonary affection is the principal cause of morbidity and mortality in patients with CF (De Boeck et al., 2005; Meyerholz et al., 2010; Olivier et al., 2012; Cutting, 2015; de Bari et al., 2018).

**FIGURE 1:**
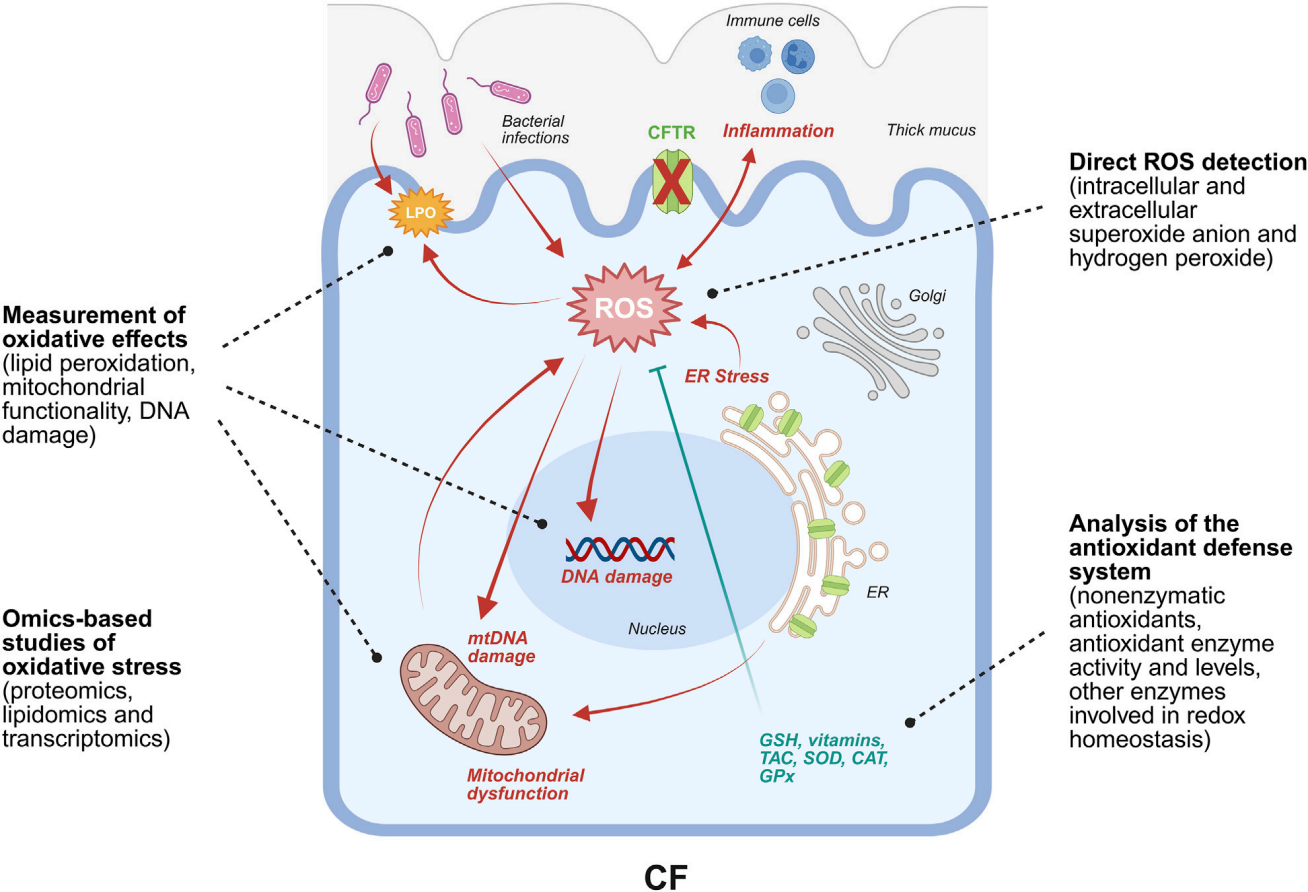
Schematic representation of the main events leading to ROS generation and accumulation in CF. Specifically, ROS generation is favored by F508del-CFTR retention in the ER that leads to ER stress, associated with the alteration of Ca^2+^ homeostasis and, consequently, with mitochondrial dysfunction. The heightened mitochondrial respiration further contributes to ROS generation. Bacterial infections promote ROS production and inflammation that, in turn, fuels oxidative stress. In fact, immune cells release ROS as a weapon to counteract pathogens. Direct consequences of elevated levels of ROS include nuclear and mitochondrial DNA damage, lipids and proteins oxidation, transcriptional regulation of genes expressing proteins involved in the antioxidant response, and modulation of the activity of antioxidant enzymes. Strategies to study oxidative stress are based on the quantification of ROS species together with the characterization of such cellular alterations. Image was drawn using BioRender (https://www.biorender.com/).

Over years, several *in vitro* and *in vivo* studies have demonstrated the establishment of a pro-inflammatory environment associated to CFTR dysfunction, especially in the lungs (De Rose, 2002; Galli et al., 2012; Cantin et al., 2015; Moliteo et al., 2022). This event happens early in life and has no apparent dependence on detectable bacterial or viral infections, which, in turn, cause an unequal intensification of the inflammatory process and its chronicity (Recchiuti et al., 2019). While the intense basal level of infiltrated neutrophils and the establishment of bacterial infections are primary contributors to the generation of ROS in CF, the underlying cellular dysfunction provides additional, intrinsic sources of oxidative stress. Specifically, the accumulation of misfolded CFTR in the endoplasmic reticulum (ER), as in the case of the F508del mutation, induces ER stress that fuels ROS production. The formation of ROS is also a consequence of defective mitochondria that, additionally, leads to the activation of the inflammasome and of the pro-inflammatory nuclear factor kappa-light-chain-enhancer of activated B cells (NF-κB). Of note is that in CF an intrinsic downregulation of the antioxidant response regulator and NF-κB antagonist Nuclear Factor Erythroid 2-related factor 2 (Nrf2) is described (Moliteo et al., 2022). Damages to membrane lipids and to nuclear and mitochondrial DNA are natural consequences of uncontrolled oxidative events. An overview of the sources of ROS and their deleterious effects on cells are depicted in Figure 1. The augmentation of oxidative stress accompanied by an impaired antioxidant machinery in CF cells has been proposed as a possible explanation for the onset of inflammation, despite it is still not clear the causal relationship. In fact, the debate still resembles the “chicken and egg paradox”, not being able to dissect what comes first. Considering the impact that inflammation exerts on lung function and disease progression, clarifying the role of oxidative stress in the pathophysiology of the disease and the identification of possible therapeutical targets within this context become imperative. While the complex relationship between CFTR dysfunction and oxidative stress, as well as the clinical outcomes of antioxidant therapies, have been discussed at length in numerous excellent reviews (Galli et al., 2012; Ciofu et al., 2019; Moliteo et al., 2022; Artusi et al., 2025), a critical and systematic analysis of the methodologies employed to generate these data is still lacking.

This review aims to systematically compile and analyze methodologies previously employed in the CF field for the characterization of oxidative stress markers. Furthermore, selected applications and outcomes of these methodologies are presented herein, with particular emphasis on highlighting potential inconsistencies arising from variations in experimental models and/or technical approaches. Subsequent sections address the direct detection of ROS, the characterization of primary oxidative process effects on proteins and lipids, alterations in gene expression, and mitochondrial functional parameters. Additionally, this review provides a comprehensive assessment of assay techniques used to investigate major non-enzymatic and enzymatic antioxidant systems, thereby offering a thorough methodological framework for researchers in this field.

## 2 CF models

The choice of the experimental model is a crucial step in research, and it must consider various factors (i.e., duration of the experiment, costs, facilities and materials required, etc.). Since several reviews on *in vivo* and *in vitro* CF models are available in the literature (Rosen et al., 2018; McCarron et al., 2021; Grubb and Livraghi-Butrico, 2022; Silva et al., 2022; Ramalho et al., 2023), we are going to give only a brief overview of the principal models discussed in the subsequent sections.

### 2.1 *In vitro* models

Immortalized cell lines are the easiest model for CF, both in terms of handling and complexity. In fact, they are easy to be cultured and possibly can divide indefinitely. Multiple human and non-human cell lines are available with epithelial or non-epithelial origins. Examples of epithelial cell lines exploited in CF research are Fisher rat thyroid (FRT) (Sheppard et al., 1994) and CF bronchial epithelial (CFBE41o-) cell lines. CFBE41o-cell line was generated from patient-derived trachea-bronchial cells transformed with SV40 large T antigen for immortalization (Ehrhardt et al., 2006). The CFBE41o-cell line is homozygous for F508del-CFTR, the most common *CFTR* mutation that causes the misfolding and premature degradation of the channel. This parental line, which intrinsically expresses minimal CFTR protein, serves as a reliable model and is frequently used as a null-background for the stable overexpression of wild-type, F508del, or other CFTR variants, enabling the study of their function and response to modulators (Bebok et al., 2005). The CFBE41o-cell line is generally compared to 16HBE14o-cell line, an immortalized human bronchial epithelial cell line endogenously expressing wild-type (WT) CFTR (Ousingsawat et al., 2011; Lasalvia et al., 2016). More recently, 16HBE14o-line has been utilized as a platform for CRISPR/Cas9-mediated gene editing to introduce specific rare CF-causing mutations (e.g., W1282X, N1303K). These isogenic, mutation-specific cell lines have become a crucial tool for the pre-clinical screening and development of mutation-specific CFTR modulators (Valley et al., 2019). Another example of human bronchial epithelial cell line is represented by IB3-1 cells, derived from a CF patient carrying oneF508del allele and one W1282X nonsense mutation allele. IB3-1 were then transduced with an adeno-associated viral vector to express a functional CFTR, thus producing an isogenic control (C38 cell line) (Silva et al., 2022; Ramalho et al., 2023).

Anyway, immortalization has some limitations like possible genome instability or alterations in gene expression that could lead to misinterpretation of the results. Therefore, validation of results in primary cells is a fundamental step. In fact, patient-derived cells may recapitulate several features of the parental organ, especially the complexity obtained with cell differentiation. Primary human bronchial epithelial cells (HBE) and nasal cells (HNE) are obtained from bronchial brushing or explants of lungs and from nasal brushing, respectively. They can be harvested in planar cultures on porous membrane filters at air-liquid interface (ALI) conditions. These primary cell models are of high translational value; in particular, data from HNE cells cultured at ALI have been shown to correlate strongly with *in vivo* clinical responses to CFTR modulators, serving as an effective pre-clinical tool to predict patient-specific drug efficacy. Furthermore, studies have established HNE cells as a reliable and less invasive surrogate for HBE cells for these functional studies (Brewington et al., 2018). Airway organoids can be derived from HBE, HNE or induced pluripotent stem cells (iPSCs). iPSC-derived airway epithelial cells can be differentiated and grown as 3D organoids that recapitulate key features of the native airway. These patient-specific organoids are particularly powerful for high-throughput screening of CFTR modulators, as they allow for testing drug responses on a patient’s unique genetic background in a renewable and scalable model system, which is especially valuable for rare mutations (Boecking et al., 2022). This model reproduces both the cellular composition and the ordered architecture of an *in vivo* airway epithelia. The invasiveness of the procedures, the complexity and the costs for their *in vitro* maintenance make them suitable limitedly for validation procedures.

*In vitro* models can also be generated from tissues acquired from CF animal models to sustain results from *in vivo* studies (McCarron et al., 2021).

### 2.2 *In vivo* models

In the last 30 years numerous animal models for CF have been developed for research purposes. Specifically, they are applied to better understand the pathophysiology of the disease, but also to test potential therapeutics. Different species have been considered like mouse (Colledge et al., 1995; Guilbault et al., 2007), rat (Dreano et al., 2019), ferret (Sun et al., 2010), pig (Rogers et al., 2008; Welsh et al., 2009), *Drosophila melanogaster* (Touré et al., 2023), zebrafish (Cafora et al., 2019; Bernut et al., 2020) and sheep (Fan et al., 2018; Viotti Perisse et al., 2021), each one with its own pros and cons.

A large number of mouse strains have been proposed and, according to McCarron et al., they can be categorized into three main groups: *(i) Cftr* null or knock out (KO) models. They recapitulate well the systemic disease with the exception of pancreatic and liver disease and spontaneous lung disease; *(ii)* mouse with *Cftr* gene modified to introduce mutations that are causative of CF in human. They recapitulate the disease but tend to be less severe; *(iii)* transgenic models like mice expressing human *CFTR,* gut-corrected mice (solved the problem of high mortality due to intestinal obstructions), and β-epithelial sodium channel (β-ENaC) mice (Gawenis et al., 2019). While mouse models are invaluable for their low cost, rapid breeding cycle, and genetic tractability, their primary limitation is the failure to spontaneously develop human-like lung disease, which is the main cause of morbidity in patients. However, they are excellent for studying the severe intestinal defects, systemic inflammation, and for screening of systemically administered drugs (Grubb and Livraghi-Butrico, 2022).

Generation of CF models with other species are mostly reached by *CFTR* KO, like in the case of rat, ferret, rabbit, pig and sheep. Moreover, for rat, pig and zebrafish CFTR was modified to introduce the F508del mutation. Models expressing the G551D *CFTR* or G551D human *CFTR* have been generated for ferret and rat, respectively (Rogers et al., 2008; Sun et al., 2008; Klymiuk et al., 2012; Navis and Bagnat, 2015; Fan et al., 2018; Dreano et al., 2019; Birket et al., 2020). *D. melanogaster* does not express CFTR but possesses an equivalent gene that has been knocked down and subsequently rescued with human CFTR expression (Kim et al., 2020).

From the phenotypical point of view rats behave like mouse strains, while pigs, ferrets and sheep manifest severe lung symptoms and they are preferred for short-term translational studies (McCarron et al., 2021). Specifically, pigs and ferrets are considered high-fidelity models because of their lung anatomy, physiology, and pathology (including spontaneous bacterial infections, inflammation, and mucus plugging) closely recapitulate the human CF condition from birth. This makes them ideal for studying early disease pathogenesis and for testing novel therapeutics like gene therapy or inhaled drugs. Their main disadvantages, however, are their high cost, long lifespan, and the need for specialized large-animal facilities. Simpler organisms like *Drosophila* and zebrafish offer unparalleled advantages for large-scale, high-throughput screening of potential CFTR modulators due to their low cost and rapid life cycle, although they cannot be used to study complex organ pathophysiology (Rosen et al., 2018; Fiorotto et al., 2019; McCarron et al., 2021; Grubb and Livraghi-Butrico, 2022). A summary of the *in vivo* models comprehensive of the main advantages and disadvantages and the principal application in CF research is reported in Supplementary Table S1.

It is evident that different *in vivo* models develop a broad spectrum of symptoms and severity according to the species, but also to the technique adopted for their generation. At the same time, also *in vitro* models could manifest differences related to their origins and generation, but also to the CFTR mutation that they carry. For these reasons, any *in vitro* or *in vivo* model of CF utilized for the study of oxidative stress or its implication in the disease pathogenesis, should be first characterized with the most appropriate techniques proposed in the following sections.

### 2.3 Biofluids from patients

Biological samples like sputum can be collected from patients with CF and exploited for research use. Whole blood, its components (plasma or serum) and circulating cells (peripheral blood lymphocytes, circulating neutrophils, freshly isolated monocytes, red blood cells) are frequently studied in the field of oxidative stress research (Regelmann et al., 1991; Benabdeslam et al., 1999; Lands et al., 1999; Lagrange-Puget et al., 2004; Yoo et al., 2014). Exhaled breath condensate (EBC) is collected by cooling exhaled air, enriched of aerosolized particles and volatile compounds contained in the breath. EBC content includes biomarkers of oxidative stress and inflammation and represents a useful source of information of the respiratory condition of the patient of origin (Spicuzza et al., 2018; Galiniak et al., 2022; Maniscalco et al., 2024).

Bronchoalveolar lavage (BAL) is an invasive procedure characterized by the infusion of a saline solution through a bronchoscope to wash the airways and the fluid is collected for subsequent analysis. BAL allows the sampling of the epithelial lining fluid (ELF), a thin layer covering the surface of alveoli, small and large airways, is the first barrier to protect lungs from external stimuli like pathogens and irritants. ELF contains low molecular weight antioxidants When analysing results from ELF studies, dilution should be considered. In fact, in the sampling process of BAL, inevitably ELF gets diluted. Strategies for correction of ELF dilution have been proposed but none of them is considered an accurate and reliable tool, making hard the comparison of data obtained from different studies and different patients (Rennard et al., 1985; Dauletbaev et al., 2004; Haeger et al., 2024).

## 3 Direct study of ROS

ROS are highly reactive chemical entities that derive from molecular oxygen during metabolic reactions. Small amounts of ROS have been proven to be beneficial for the cells, acting as mediators of signalling pathways (Sarbassov and Sabatini, 2005; Waghray et al., 2005; McCubrey et al., 2006; Reznick et al., 2007; Fukai and Ushio-Fukai, 2011). On the contrary, high amounts are deleterious for the biology and physiology of cells. The alteration of the balance between the ROS amount and the antioxidant system in favour of the former leads to oxidative stress. Thus, a direct measurement of ROS can be indicative of the oxidative stress condition of the model/sample analysed. The most common tool applied for direct detection of ROS consists of fluorogenic probes, whose fluorescence increases, is bleached or shifts in maximum peak in response to a target molecule or event. In general, detection methods are based on the use of fluorescence microscopy (Abdalla et al., 2017; Zhang et al., 2022), flow cytometry (Kauffman et al., 2016) or spectrofluorometer (Steenbergen et al., 1997; Alhasan and Njus, 2008). Commercially available kits for ROS detection are also available, but they should be used only when their mechanism is explicated, and it is highly recommended to confirm results from kits with other techniques. A comprehensive overview of all the techniques described in Sections 3, 4, 6 is summarized in Supplementary Table S2.

### 3.1 Superoxide anion

#### 3.1.1 Ferricytochrome c reduction assay (SOD-inhibitable superoxide quantification)

Extracellular superoxide quantification can be performed exploiting the ability of the superoxide to reduce ferricytochrome c. Ferricytochrome c reduction can be monitored measuring absorbance at 550 nm, that corresponds to the absorbance maximum of the reduced form of this protein (Fridovich, 1970; Quick et al., 2000). So, the higher the amount of superoxide, the higher the signal recorded. To determine the nmoles of superoxide produced, it should be run an identical sample that contains superoxide dismutase (SOD) for every experimental condition. Then, the difference in absorbances in the absence and presence of SOD corresponds to superoxide contribution. It is recommended to measure the two samples in parallel because also other molecules can reduce ferricytochrome c (i.e., glutathione (GSH) and ascorbate). Moreover, catalase (CAT) can be included to eliminate unwanted hydrogen peroxide (H_2_O_2_)-mediated reactions from the assay (Quick et al., 2000; Nauseef, 2013).

#### 3.1.2 Dihydroethidium and MitoSOX

Dihydroethidium (DHE), also known as hydroethidine (HE), is a fluorogenic probe. Its superoxide-mediated oxidation generates the fluorescent product 2-hydroxyethidium (2-E^+^OH) (λ_excitation_ = ∼ 500 nm, λ_emission_ = ∼ 600 nm), while other ROS or reactive nitrogen species (RNS) do not form the same fluorescent product when reacting with DHE (Zhao et al., 2003; Zielonka and Kalyanaraman, 2010). The increase in fluorescence intensity is directly proportional to superoxide amount. The specificity of the detection can be confirmed by exploiting the competition between DHE and SOD (Michalski et al., 2013).

MitoSOX Red is a derivative of HE, synthesized to detect superoxide in mitochondria. The mitochondrial localization is driven by a hexyl triphenylphosphonium cation, covalently bonded to DHE (Robinson et al., 2006).

#### 3.1.3 Superoxide-mediated adrenochrome generation

Another method applied to investigate superoxide levels is based on the ability of the anion generated during the xanthine oxidase-mediated reaction to oxidize epinephrine. This event initiates a sequence of reactions that terminates with the formation of the coloured product adrenochrome. The change of absorbance at 485 nm as a consequence of adrenochrome generation is indicative of superoxide formation (Chen and Thakker, 2002; Alhasan and Njus, 2008).

### 3.2 Hydrogen peroxide

#### 3.2.1 Dichlorofluoresceine

Frequently MitoSOX measurements are associated with staining with 5-(and -6)-carboxy-2′,7′-dichlorodihydrofluoresceine diacetate (DCFH-DA) for the evaluation of H_2_O_2_ basal content. Specifically, DCFH-DA is a membrane permeable non-fluorescent probe that diffuses into the cells, and it is activated into 2′,7′-dichlorodihydrofluoresceine (DCFH) by esterase-mediated deacetylation. The removal of the acetate groups generates a polar molecule that remains trapped into the cell. Then, DCFH is oxidized to DCF by intracellular H_2_O_2_, generating the fluorophore DCF (λ_excitation_ = 488 nm, λ_emission_ = 530 nm). Therefore, following H_2_O_2_-mediated oxidation of DCF, measurements of the fluorescence intensity can be predictive of the H_2_O_2_ produced by the cells (Ubezio and Civoli, 1994; Reiniers et al., 2017).

#### 3.2.2 Peroxidase-mediated assays

Extracellular H_2_O_2_ can be quantified by exploiting its ability to oxidize susceptible probes in the presence of a peroxidase (Nauseef, 2013).

Scopoletin is a fluorescent agent that is bleached when oxidized, whereas HVA, phenol red, and ADHP exhibit increased fluorescence upon oxidation. HVA is a substituted phenol that, when oxidized, produces a fluorescent dimer (λ_ex_ = 321 nm, λ_em_ = 421 nm) (Thannickal and Fanburg, 1995). HRP-mediated oxidation of phenol red by H_2_O_2_ results in the formation of a compound measurable at 610 nm absorbance, with a linear relationship between H_2_O_2_ concentration and absorbance (Pick and Keisari, 1980).

ADHP is a colorless, highly stable, and nonfluorescent substrate that, upon oxidation, becomes a highly fluorescent molecule named resorufin (λ_ex_ = 561 nm, λ_em_ = 585 nm). Although ADHP oxidation by other types of ROS has been described, this occurs at a lower yield than H_2_O_2_-mediated oxidation. Moreover, the ADHP-induced increase in fluorescence is inhibitable by CAT, confirming its specificity for H_2_O_2_ detection. Compared to scopoletin, ADHP offers advantages including (i) a low background that barely changes over time and (ii) higher sensitivity due to its high fluorescent yield (Mohanty et al., 1997).

Another fluorescent probe to measure H_2_O_2_ is the 2,2′-dihydroxy-biphenyl-5,5′ diacetate, the stable dimer generated from the oxidation of the p-hydroxyphenylacetic acid (pHPA) by the Complex I. This, in turn, had been oxidised by the H_2_O_2_-consuming HRP (Schick et al., 1997).

### 3.3 Results: direct detection of ROS in CF

#### 3.3.1 Superoxide anion

Early investigations into the oxidative burst mechanisms in CF revealed notable differences in immune cell activity, even among carriers of a single CFTR mutation (heterozygotes). Utilizing the ferricytochrome c reduction assay for SOD-inhibitable superoxide quantification, Regelmann et al. observed that monocytes isolated from CF heterozygotes, when stimulated with concanavalin A, exhibited a significantly higher release of superoxide compared to monocytes from healthy controls after a 3-min measurement period. This heightened oxidative burden extends beyond immune cells to the airway epithelial cells, which are primary sites of CFTR dysfunction (Regelmann et al., 1991). Studies employing CFBE41o-cells have corroborated these findings, demonstrating elevated superoxide levels.

For instance, ferricytochrome c reduction and adrenochrome assay measured significantly higher levels of superoxide anion in CFBE41o-cells compared to WT controls in two independent studies (Atlante et al., 2016; de Bari et al., 2018). Moreover, MitoSOX Red probe, in combination with the mitochondrial Complex I inhibitor rotenone, pinpointed this mitochondrial complex as a principal source of the augmented superoxide production in these CF cells (Atlante et al., 2016). Furthermore, treatment with Lumacaftor, a CFTR corrector facilitating the trafficking of misfolded F508del-CFTR protein, led to a reduction of superoxide levels in CFBE41o-cells, suggesting that while Lumacaftor primarily addresses CFTR protein processing, its therapeutic efficacy might also stem from mitigating oxidative damage and subsequent inflammatory responses (de Bari et al., 2018). Analogously, it was discovered that CFTR corrector therapy can modulate a metabolic shift detected with MitoSOX as an elevation of mitochondrial ROS production associated with increased mitochondrial respiration (Jarosz-Griffiths et al., 2024).

Exposure of primary HBE cells to *Pseudomonas aeruginosa* quinolone resulted in a significant increase in superoxide production (Abdalla et al., 2017). Another research using MitoSOX Red has specifically identified augmented mitochondrial superoxide production in CF macrophages, where altered mitochondrial ROS levels are implicated in diminished bacterial clearance and heightened inflammation (Hamilton et al., 2021). Studies utilizing MitoSOX Red have shown increased mitochondrial superoxide levels in IB3-1 cells and primary cells expressing F508del-CFTR, particularly following infection with *P. aeruginosa*, thereby suggesting a connection between bacterial challenge and mitochondrial oxidative stress in CF airways (Rimessi et al., 2015; 2020). Notably, earlier research using MitoSOX also demonstrated significantly elevated mitochondrial ROS in CFTR-deficient IB3-1 cells compared to control cells (Velsor et al., 2006). Beyond airway cells and bacterial interactions, DHE-based investigations have indicated increased general intracellular superoxide in the pancreas of CF pigs, a finding linked to endothelial dysfunction within the CF context (O’Malley et al., 2022).

#### 3.3.2 Hydrogen peroxide

Investigations into H_2_O_2_ and related ROS have employed various methodologies, including the use of DCFH-DA and its derivatives. The assessment of general intracellular ROS using DCFH-DA has produced varied outcomes contingent on cell type and experimental context. For example, DCFH-DA assay measured an increased oxidative burst in peripheral blood neutrophils from CF patients (Frühwirth et al., 1998), while no significant differences in total intracellular ROS between CF and WT mouse macrophages were detected at baseline or following bacterial challenge (Hamilton et al., 2021). Conversely, DCFH-DA oxidation indicated increased intracellular H_2_O_2_ levels in CFTR-deficient IB3-1 cells (Velsor et al., 2006), although no differences in bulk oxidative capabilities, were noted in homozygous F508del-CFTR nasal epithelial cells (Schwarzer et al., 2007). Methodological nuances concerning probe application and detection further influence the interpretation of these findings (Clauzure et al., 2021).

Peroxidase-mediated assays offer alternative approaches to quantify H_2_O_2_. Clinical research has utilized techniques like the pHPA coupled with HRP to detect elevated H_2_O_2_ levels in the EBC of children with CF during acute pulmonary infections, with these levels decreasing following antibiotic treatment (Jöbsis et al., 2000). In cellular models, the Amplex Red^®^ assay can directly quantify accumulated H_2_O_2_, as shown in studies measuring its release rate into the apical solution of CF HNE cells grown on permeable supports after viral infection (Schwarzer et al., 2007). While the primary focus remains on CF-relevant systems, techniques for measuring intracellular ROS have also been applied in studies investigating the effects of compounds on oxidative status in other cell types, such as platelets, in inflammatory contexts, highlighting the broader applicability of these methodologies in understanding redox biology (Gziut et al., 2013). These diverse approaches underscore the varied strategies employed to quantify and comprehend the role of H_2_O_2_ and related oxidants in the complex pathology of CF and associated inflammatory processes.

## 4 Study of oxidative effects

### 4.1 Lipid hydroperoxides

#### 4.1.1 Thiobarbituric acid reactive substances assay

The Thiobarbituric Acid Reactive Substances (TBARS) assay is an old but commonly used test to study lipid peroxidation by exploiting the product malondialdehyde (MDA) as a marker of the reaction. This assay is based on the ability of the MDA to react with the thiobarbituric acid (TBA) under acidic condition, leading to the formation of MDA-TBA2 adducts called TBARS. TBARS are characterized by a red color pigment, that can be measured both using visible wavelength spectrophotometry at 532 nm and fluorescence (λ_excitation_ = 370 nm, λ_emission_ = 420 nm). Actually, this method presents some limitations: *(i)* not all lipid peroxidation reactions lead to the formation of MDA; *(ii)* some molecules other than MDA can react with TBA, producing adducts with similar absorption to TBARS; *(iii)* MDA is not generated exclusively through lipid peroxidation; *(iv)* the experimental conditions required for MDA reaction with TBA (high temperature and low pH) may cause artefactual formation of lipid peroxidation products, eventually producing misleading data (Janero et al., 1990; Jentzsch et al., 1996). It is reasonable to suggest that TBARS test is associated with data from other methods to study lipid peroxidation, or that the implementation of TBARS test proposed by Jentzsch in 1996 is adopted. Specifically, to avoid lipid peroxidation reactions dependent on experimental settings, authors proposed to exclude oxygen or add high amounts of butylhydroxytoluene (BHT) (Jentzsch et al., 1996).

#### 4.1.2 FOX1 assay

Hydroperoxides can be estimated with the ferrous ion oxidation xylenol orange (FOX) methods. Two version of this method are described: FOX1 is used for the determination of low levels of lipid hydroperoxides in aqueous phase, while FOX2 is applied in case of lipid phase. FOX1 is described as more sensitive than FOX2 (Wolff, 1994). Briefly, in dilute acid, lipid hydroperoxides oxidises ferrous to ferric ion. Xylenol orange is a fluorochrome acid dye with high selective chelating properties for ferric ions. Their interactions produce a coloured complex that can be detected with a spectrophotometer at 560 nm. Thus, it is possible to indirectly measure hydroperoxide concentration by monitoring ferric ions generation (Banerjee et al., 2003).

#### 4.1.3 Cis-parinaric acid

Cis-parinaric acid (PnAc) is a naturally fluorescent fatty acid, applied as a structural analogue of membrane lipids. Its chemical and physical properties make it appropriate as a probe for lipid peroxidation. In fact, when excited at ∼ 320 nm, this chromophore provides for a natural fluorescence at ∼ 420 nm, which is decreased by oxidative stimuli. Thus, the greater lipid peroxidation, the less fluorescence is detected (Laranjinha et al., 1992; Steenbergen et al., 1997).

#### 4.1.4 BODIPY™ 581/591 C11 and MitoPerOx

BODIPY™ 581/591 C11 undecanoic acid is a lipid peroxidation sensor that localizes to membranes. Upon its oxidation, a shift of the fluorescence emission happens. Specifically, to the reduced form corresponds an emission peak at ∼590 nm, while to the oxidised form the peak is shifted to ∼510 nm. This assay allows a ratiometric analysis of membrane lipid oxidation (Drummen et al., 2002; Wiernicki et al., 2020; Stockert, 2021).

A derivative from BODIPY™ 581/591 C11 called MitoPerOx has been developed in 2012 to detect mitochondrial lipid hydroperoxides. In this probe the BODIPY fluorophore was conjugated via a dienyl group to a phenyl group, and, similarly to MitoSOX, the specificity for the inner membrane of the mitochondria (facing the matrix) is given by a triphenylphosphonium lipophilic cation, whose uptake is driven by the mitochondrial membrane potential. Analogously to C11-BODIPY, probe oxidation induces a shift of the fluorescence emission peak from ∼590 to ∼520 nm (Prime et al., 2012; Oh et al., 2022).

### 4.2 Mitochondrial functionality

#### 4.2.1 Seahorse

Oxidative stress is characterized by the accumulation of ROS, which can induce damages to the mitochondrial respiratory chain, influence Ca^2+^ homeostasis, and impair mitochondrial functionality. The Seahorse XF (Extracellular Flux) technology is an advanced analytical platform designed for real-time, label-free, and non-destructive evaluation of cellular bioenergetics. This system enables the simultaneous measurement of oxygen consumption rate (OCR), reflecting mitochondrial oxidative phosphorylation, and extracellular acidification rate (ECAR), indicative of glycolytic activity. Together, these metrics delineate the two principal adenosine triphosphate (ATP)-generating pathways in viable cells, which may be affected by oxidative stress.

The methodology employs specialized multi-well plates seeded with adherent or suspended cell cultures. A sensor cartridge equipped with biosensors for dissolved oxygen and hydrogen ion concentration is positioned above the wells, establishing transient, isolated microenvironments. Within these microenvironments, fluctuations in oxygen tension and pH levels - driven by cellular metabolic processes - are continuously monitored. Through timed injections of pharmacological modulators (e.g., metabolic inhibitors, activators or substrates), the assay facilitates a comprehensive investigation of mitochondrial and glycolytic functionality. Key parameters assessed include basal and maximal respiratory capacity, ATP-linked respiration, spare respiratory capacity, basal glycolysis, and glycolytic reserve. This dynamic, multi-parametric approach yields a detailed bioenergetic profile, enabling rigorous characterization of cellular metabolic adaptations under controlled experimental conditions (Ferrick et al., 2008).

### 4.3 DNA damage detection

DNA damage is one of the major consequences of oxidative stress, including base modifications, strand breaks, and base loss. Guanine has the lowest redox potential, thus making it readily oxidised. Among its oxidation products there are the 8-hydroxy-2′-deoxyguanosine (8-OHdG) and the 8-oxo-7,8-dihydroguanine (8-oxoGua) (Zhang et al., 2013).

8-OHdG is considered one of the most common DNA modifications in case of oxidative stress conditions and 8-oxoGua directly derives from it. 8-OHdG can be excised from the DNA during repair mechanisms, thus being released in the blood stream and subsequently be excreted in the urine, patients-derived biofluids where it can be detected (Lunec et al., 2002). Both can be detected with high performance liquid chromatography (HPLC) with electrochemical detection or gas chromatograph-mass spectrometry (GS-MS) with selective ion monitoring (Lin et al., 2004). Enzyme-linked immunosorbent assay (ELISA) is commonly used for the quantification of guanosine oxidation products, but chromatographic techniques are considered more reliable (Yin et al., 1995; Rossner et al., 2016; Drake et al., 2019).

Strand breaks can be measured by the alkaline unwinding procedure, based on the discovery that double-stranded DNA (dsDNA) unwind in an alkaline solution from free ends. The amount of residual dsDNA is proportional to the number of strand breaks. The separation of single-stranded (ssDNA) and dsDNA are measured by hydroxyapatite chromatography, followed by detection of DNA with DNA binding dyes (Bolcsfoldi, 1995; Moreno-Villanueva et al., 2009; Nicolai et al., 2021). An alternative is offered by the comet assay. Specifically, the higher the number of breaks, the higher their capacity of relaxing the DNA supercoiling thus allowing its migration in an electrophoresis gel. The percentage of DNA in the tail of the comet-like image of migration reflects break frequency (Collins et al., 2023).

### 4.4 Results: study of oxidative events in CF

#### 4.4.1 Lipid hydroperoxides

Markers of lipid peroxidation, notably TBARS and MDA, are crucial indicators of oxidative damage to cellular membranes and are frequently evaluated in CF to assess the impact of oxidative stress. A substantial body of research reports elevated TBARS and MDA levels in diverse biological samples from CF patients compared to healthy controls. These include plasma (Brown and Kelly, 1994; Portal B. et al., 1995; Back et al., 2004; Bennemann et al., 2022), and white blood cells, even in patients considered clinically stable, indicating a chronic inflammatory and oxidative state (Olveira et al., 2013). Conversely, one study noted initially lower MDA levels in CF patients that increased with age and clinical deterioration and observed an increase in lipid peroxidation (TBARS and FOX1) following antibiotic administration, highlighting potential variability influenced by disease stage and treatment (Lagrange-Puget et al., 2004). In CF animal models, increased TBARS have been demonstrated in the lung tissue of *Cftr*-KO mice, reflecting localized oxidative damage within affected organs (Velsor et al., 2001), although TBARS in BAL fluid (BALF) were below detection limits in both *Cftr*-KO and WT mice.

The FOX1 method was notably employed by Hull and colleagues and revealed a significant elevation in lipid hydroperoxide concentrations in the ELF of CF patients exhibiting inflammation, compared to both the control group and the CF group without inflammation. Crucially, lipid hydroperoxide levels in CF patients without inflammation were similar to those in healthy controls (Hull et al., 1997). This provided direct evidence of increased lipid peroxidation in the CF airway and strongly suggested that this elevation is a consequence of the pulmonary inflammation characteristic of CF, rather than a direct, inflammation-independent outcome of the primary CF defect.

PnAc enabled the demonstration of an exacerbated lipid peroxidation in the mitochondrial membranes of CFBE41o-cells compared to 16HBE14o-cells, thereby adding to the cumulative evidence of increased oxidative damage at the mitochondrial level in CF (Atlante et al., 2016).

Furthermore, a pivotal study by Maniam et al. demonstrated the increased susceptibility to ferroptosis of IB3-1cells in comparison to control epithelial cells. In fact, BODIPY™ 581/591 C11 revealed heightened levels of lipid peroxidation in the CF cell line and provided compelling evidence that CF airway epithelial cells are predisposed to accumulate lipid peroxides, a key driver of ferroptosis (Maniam et al., 2021).

Overall, the consistent finding of elevated lipid peroxidation markers across various studies and sample types provides robust evidence of heightened oxidative stress in CF, contributing to cellular dysfunction and disease progression.

#### 4.4.2 Mitochondrial functionality in CF

Immune cells, such as macrophages and neutrophils, exhibit significant metabolic reprogramming in the context of CF, profoundly influencing their inflammatory responses and ability to effectively clear pathogens. Seahorse XF analysis has proven instrumental in comprehensively characterizing these altered metabolic profiles in CF immune cells. For instance, investigations into defective immunometabolism in CF macrophages have utilized the Seahorse Extracellular Flux analyzer, revealing impaired oxygen consumption, both at baseline and following infection with challenging pathogens like *Burkholderia cenocepacia* (Hamilton et al., 2021). Further applying Seahorse extracellular flux assays, studies have demonstrated that the heightened mitochondrial respiration observed in CF cell lines, including HBE cells expressing CFTR^F508del/F508del^, can be remarkably normalized by triple CFTR modulator therapy through mechanisms involving calcium (Jarosz-Griffiths et al., 2024). Additionally, utilizing Seahorse XF assays to delve into the immunometabolism of monocyte-derived macrophages (MDMs), research has shown that exposure to secondhand e-cigarette vapor significantly impacts macrophage metabolic profiles in both CF and non-CF cells; notably, vape exposure blunted macrophage basal respiration, an effect partially rescued by CFTR modulator therapy (ETI), while concurrently increasing glycolytic metabolism, a change unaffected by ETI treatment (Wisniewski et al., 2025).

#### 4.4.3 DNA damage detection in CF

In CF, increased amount of 8-OHdG was measured both in nuclear DNA (Velsor et al., 2001; Velsor et al., 2001) and mitochondrial DNA (Velsor et al., 2006) extracted from lung tissues of *Cftr*-KO mice and compared to WT. Analogously, urinary level of 8-OHdG was significantly higher in CF children than in age-matched controls and this event was independent of clinical status (Brown et al., 1995).

Another application consists in the measurement of 8-oxoGua in the DNA of *P. aeruginosa* strains. From these studies it emerged that hypermutable *P. aeruginosa* strains have more 8-oxoGua upon exposure to polymorphonucleates (PMN) and this mechanism is proposed to be linked to an increased antibiotic resistance. For this reason, authors suggest an antioxidative therapy for patients with CF to diminish oxidative stress, thus reducing the risk of developing antibiotic resistance (Ciofu et al., 2005; Mandsberg et al., 2009).

## 5 Omics to study oxidative stress

### 5.1 Proteomics

Proteomics uniquely illuminates oxidative stress by enabling precise quantification of antioxidant enzymes, detection of oxidative post-translational modifications, and analysis of redox-sensitive pathways. In CF, oxidative stress functions as a key driver of tissue injury, chronic inflammation, and CFTR dysfunction. Characterizing the CF “oxidative stress proteome” remains essential for identifying therapeutic targets and evaluating treatment efficacy. Sample selection critically influences research outcomes, with airway-derived specimens like sputum providing non-invasive inflammatory insights (Sagel et al., 2012), while BALF accesses deeper airways despite invasiveness limitations (Gharib et al., 2010; Kruk et al., 2024). Nasal samples offer accessibility but incompletely represent lower airway oxidative stress (Jeanson et al., 2014), whereas EBC presents biomarker potential despite standardization challenges (Causer et al., 2020; Yoo et al., 2024). Systemic specimens enable minimally invasive assessment of oxidative damage markers (Causer et al., 2020; Fentker et al., 2024), complemented by sweat proteomics identifying CFTR-related biomarkers (Burat et al., 2022). *In vitro* models isolate specific mechanisms like mitochondrial GSH depletion (Velsor et al., 2006) and Nrf2 dysfunction (Chen et al., 2008) under controlled conditions. Rigorous sample handling protocols remain imperative across methodologies to preserve redox-sensitive modifications and ensure data integrity (Fentker et al., 2024).

#### 5.1.1 Gel-based proteomics

Two-dimensional polyacrylamide gel electrophoresis (2D-PAGE) separates complex protein mixtures by isoelectric point and molecular weight, enabling analysis of thousands of protein spots in a single gel (Kavallaris and Marshall, 2005). This technique has contributed to CF research through BALF proteome studies, revealing low molecular weight protein differences between CF patients and controls (von Bredow et al., 2001). Its enhanced version, two-dimensional difference gel electrophoresis (2D-DIGE), allows simultaneous quantitative comparison of multiple samples using distinct fluorescent labels, as demonstrated in CF serum proteomics investigations (Charro et al., 2011). Despite these historical contributions, gel-based approaches exhibit notable limitations that have prompted their progressive replacement by direct mass spectrometry (MS)-based methodologies for oxidative stress research. These traditional techniques are labor-intensive with restricted dynamic range for detecting low-abundance proteins and provide suboptimal resolution for proteins with extreme molecular weights or high hydrophobicity, including CFTR (Charro et al., 2011). Contemporary CF research has consequently shifted toward MS-centric methodologies offering superior sensitivity, throughput, and capabilities for identifying specific post-translational modifications essential for comprehensive oxidative stress analysis.

#### 5.1.2 MS-based proteomics: shotgun proteomics (LC-MS/MS, MudPIT)

MS has established itself as the preeminent analytical platform in modern proteomics research, offering exceptional sensitivity, specificity, and throughput for protein identification and quantification. Shotgun proteomics, primarily utilizing liquid chromatography coupled to tandem mass spectrometry (LC-MS/MS), provides a robust methodology for comprehensive protein identification in complex biological matrices. This “bottom-up” approach begins with enzymatic protein digestion (typically using trypsin) to generate peptides, which undergo liquid chromatographic separation before mass spectrometric analysis. The analytical workflow involves initial measurement of peptide mass-to-charge ratios (m/z) in MS1 scans, followed by fragmentation of selected peptides to produce tandem mass spectra (MS/MS) containing sequence information, which are subsequently matched against protein databases for identification (Nimer and Abdel Rahman, 2023). Multidimensional Protein Identification Technology (MudPIT) represents an advanced shotgun proteomics implementation, incorporating multiple orthogonal chromatographic separation stages prior to MS analysis, significantly enhancing proteome coverage through improved peptide mixture resolution (Rauniyar et al., 2014).

#### 5.1.3 MS-based proteomics: quantitative proteomics strategies (label-free, SILAC, iTRAQ, TMT)

Quantitative proteomics enables determination of relative or absolute protein abundance variations across distinct biological conditions, utilizing either label-free or label-based approaches.

*Label-free quantification (LFQ)* compares protein abundance based on MS signal intensity or spectral counts without isotopic labeling (Li et al., 2012). This methodology offers simplified sample preparation and versatility across various sample types, providing extensive proteome coverage. LFQ has been widely implemented in CF research, including investigations of cellular models (Rauniyar et al., 2014), BALF metaproteomics (Kruk et al., 2024), and plasma proteomics in analogous fibrotic pulmonary conditions characterized by oxidative stress (Saraswat et al., 2020). However, LFQ may exhibit lower quantitative accuracy and reproducibility compared to label-based techniques, particularly when measuring subtle alterations in oxidative stress markers or antioxidant enzymes (Li et al., 2012).

*Stable Isotope Labeling with Amino Acids in Cell Culture (SILAC)* involves metabolic labeling where cells incorporate “light” or “heavy” (stable isotope-enriched) essential amino acids during protein synthesis. Samples from different conditions are combined early in processing, with relative protein abundance determined by heavy-to-light peptide signal ratios (Peters-Hall et al., 2015). SILAC delivers high accuracy in cell culture experiments by minimizing downstream processing variability (Harsha et al., 2008), but remains primarily limited to metabolically active, culturable cells, constraining its applicability for direct patient sample analysis.

*Isobaric labeling techniques* including iTRAQ (isobaric Tags for Relative and Absolute Quantitation) and TMT (Tandem Mass Tags) involve chemical tagging of peptides post-digestion. These tags possess identical masses, enabling co-elution during chromatography and identical m/z in MS1 scans. Upon fragmentation, each tag releases a unique “reporter ion” whose intensity reflects the peptide’s original abundance (Li et al., 2012). These methods allow multiplexing of 8–16+ samples simultaneously, enhancing throughput and statistical power through concurrent analysis of multiple conditions. However, they may experience “ratio compression” from interfering peptides, potentially underestimating true protein abundance fold changes.

Selection among these quantitative approaches depends on experimental design, sample characteristics, required analytical depth, and quantitative precision needs. SILAC proves optimal for investigating oxidative stress mechanisms in controlled cell culture models, while TMT or LFQ may be preferred for larger patient cohorts or clinical samples, considering their respective strengths in quantifying redox-related proteins in CF.

#### 5.1.4 MS-based proteomics: targeted proteomics (MRM/SRM) for biomarker validation

Targeted proteomics, predominantly utilizing Multiple Reaction Monitoring (MRM) or Selected Reaction Monitoring (SRM) mass spectrometry, represents a hypothesis-driven methodology for precise and sensitive quantification of pre-selected proteins through their representative peptides. Unlike discovery-based approaches, MRM/SRM selectively monitors specific precursor ion-to-fragment ion transitions unique to target peptides (McShane et al., 2014). This high selectivity enables quantification within complex biological matrices with minimal sample fractionation. Quantitative accuracy is enhanced through incorporation of stable isotope-labeled synthetic peptides as internal standards (Liquid Chromatography-Stable Isotope Dilution-MRM MS or LC-SID-MRM MS), a methodology successfully applied for absolute quantification of CFTR protein using signature peptides as full-length protein surrogates (McShane et al., 2014). Targeted proteomics serves a critical function in translational research, bridging discovery-phase findings with clinical applications. Following identification of candidate oxidative stress biomarkers through discovery proteomics, MRM/SRM provides robust, reproducible, and high-throughput quantification essential for validation in larger clinical cohorts. This crucial validation determines the utility of potential oxidative stress biomarkers for CF diagnosis, patient stratification, disease progression monitoring, and therapeutic response assessment. The capability for absolute quantification, demonstrated for CFTR (McShane et al., 2014), provides particular value for establishing precise physiological or pathological concentrations of key proteins involved in oxidative stress responses.

#### 5.1.5 MS-based proteomics: redox proteomics

Redox proteomics provides powerful tools for investigating oxidative stress molecular impact in CF by identifying and quantifying proteins with specific oxidative or nitrosative post-translational modifications (PTMs). Protein carbonylation, an irreversible PTM introducing carbonyl groups, serves as a critical biomarker of severe oxidative protein damage in CF. These modifications result from direct ROS attack on amino acids (proline, arginine, lysine, threonine) or indirectly via reactive aldehydes from lipid peroxidation (MDA, 4-hydroxynonenal), or through reactions with reducing sugars and advanced glycation end-products (Pinzaru et al., 2023). With elevated protein carbonyls documented in CF patients (Causer et al., 2020), identifying specifically affected proteins is essential for understanding functional consequences. Traditional detection employs 2,4-dinitrophenylhydrazine (DNPH) derivatization, forming stable adducts quantifiable spectrophotometrically or via anti-DNPH antibody Western blotting (Madian and Regnier, 2010). For proteomic identification, DNPH derivatization can be coupled with 2D-PAGE and MS. Advanced approaches utilize biotin hydrazide or other tagged hydrazides for carbonyl derivatization, enabling avidin-based affinity chromatography enrichment before LC-MS/MS identification of modified proteins and specific carbonylation sites (Madian and Regnier, 2010). Profiling the “carbonylome” in CF provides a molecular fingerprint of irreversible oxidative damage, revealing vulnerable cellular pathways and potential therapeutic targets (Starosta et al., 2006). Cysteine thiols are highly susceptible to redox-based PTMs like S-nitrosylation and S-glutathionylation, which are critical for cellular signaling and stress responses. S-nitrosylation involves covalent attachment of nitric oxide (NO) to cysteine thiols, forming reversible S-nitrosothiols (SNOs) (Kimura, 2020). While essential for physiological signaling, excessive NO production in CF airway inflammation can dysregulate S-nitrosylation, contributing to pathology (Kimura, 2020). Proteomic identification typically employs “biotin-switch” assays or differential labeling techniques, followed by enrichment and MS identification (Wolhuter and Eaton, 2017). S-glutathionylation forms mixed disulfide bonds between protein cysteine thiols and GSH (Townsend, 2007), serving protective, regulatory, and signaling functions. In CF, CFTR dysfunction compromises GSH transport and homeostasis, altering S-glutathionylation patterns (Pinzaru et al., 2023). Proteomic approaches for S-glutathionylated protein identification involve trapping glutathionylated peptides or using isotopically labeled GSH derivatives for enrichment before MS-based identification and quantification (Harsha et al., 2008).

#### 5.1.6 MS-based proteomics: advanced techniques for studying protein oxidation

Redox proteomics continues to advance, offering increasingly detailed insights into protein structure, dynamics, and modifications under oxidative stress. Fast Photochemical Oxidation of Proteins (FPOP) stands out as an innovative MS-based foot printing technique utilizing highly reactive hydroxyl radicals to map solvent-accessible protein surfaces (McKenzie-Coe et al., 2021). In FPOP, hydroxyl radicals are generated on a microsecond timescale via laser-induced photolysis of H_2_O_2_. These radicals rapidly and irreversibly modify solvent-exposed amino acid side chains. Subsequent MS analysis identifies the sites and extent of these modifications, providing residue-level resolution of protein conformation, ligand binding sites, and interaction interfaces. FPOP is exceptionally suited for studying dynamic processes like protein folding and conformational changes, as its rapid labeling kinetics can “capture” transient structural states (Johnson et al., 2019). A significant recent advancement, In-Cell FPOP of Membrane Proteins (IC-FPOMP), enables foot printing of integral membrane proteins, such as CFTR, within their native live-cell environment. This addresses the challenge of structurally studying membrane proteins. Although specific applications of FPOP to CF-related oxidative damage are not yet extensively documented, its capabilities are highly promising. Since hydroxyl radicals are key damaging species in biological oxidative stress, FPOP can mimic and map proteins’ most susceptible sites to this oxidative attack. This could be instrumental in investigating how the chronic oxidative environment in CF alters the conformation of CFTR or other critical airway proteins, or how CFTR mutations affect protein structure and solvent accessibility, potentially predisposing them to oxidative damage (Johnson et al., 2019; Sun et al., 2025). While current redox proteomics in CF often focuses on identifying *which* proteins are modified (e.g., carbonylated, glutathionylated), techniques like FPOP offer the potential to delve deeper into *how* these modifications occur - identifying specific residues targeted by ROS - and how these modifications, or the oxidative environment itself, impact protein structure, dynamics, and interactions (Johnson et al., 2019; Sun et al., 2025).

Supplementary Table S3 summarizes the proteomic methodologies discussed, highlighting their principles and applications in CF oxidative stress research.

#### 5.1.7 Results: proteomics analysis in CF

##### 5.1.7.1 Proteomic findings on oxidative stress in CF

Proteomic investigations have provided substantial insights into the mechanisms and consequences of oxidative stress in CF. These studies have identified specific biomarkers of oxidative damage, elucidated the dysregulation of antioxidant defense systems, mapped connections between oxidative stress and inflammatory pathways, and began to explore the influence of CFTR genotype and modulator therapies on the redox landscape of CF.

###### 5.1.7.1.1 Specific carbonylated proteins

Protein carbonylation is a stable and irreversible marker of oxidative protein damage, and its levels are known to be elevated in individuals with CF, indicative of a significant systemic and localized oxidative burden (Pinzaru et al., 2023). This increase was evident even in patients with normal lung function and correlated directly with neutrophilic inflammation and inversely with pulmonary function (Starosta et al., 2006). While global measurements confirm this increase, large-scale “carbonylome” studies specifically from CF patient airway samples are still emerging. However, in Idiopathic Pulmonary Fibrosis (IPF), a chronic lung disease that shares some pathological features with CF, including fibrosis and significant oxidative stress, plasma proteomics has identified haptoglobin-related protein as a potential biomarker, being involved in antioxidant responses, alongside evidence of dysregulated oxidative pathways (Saraswat et al., 2020).

##### 5.1.7.2 Alterations in thiol-redox proteome (S-glutathionylated, S-nitrosylated proteins)

GSH depletion combined with chronic oxidative and nitrosative stress in CF creates favorable conditions for thiol S-glutathionylation and S-nitrosylation. Mass spectrometry studies have quantified glutathionylated proteins (GSSP) in CF airways, revealing an inherent GSH deficiency independent of oxidation status. Despite low GSH levels, GSSP was significantly elevated in CF children with pulmonary infections, correlating with increased bronchiectasis risk. This GSH deficiency impairs both antioxidant responses and regulation of S-glutathionylation/deglutathionylation cycles (Dickerhof et al., 2017). S-nitrosoglutathione (GSNO), an endogenous S-nitrosothiol in airway lining fluid, functions as a significant signaling molecule (Gaston et al., 1993) and enhances both wild-type and F508del-CFTR expression and function (Zaman et al., 2001; Chen et al., 2006; Zeitlin, 2006). At physiological or slightly elevated concentrations, GSNO improves CFTR biogenesis via both transcriptional regulation (increasing SP1/SP3 transcription factors) and post-translational modifications. Conversely, significantly higher GSNO concentrations (10–100 fold above normal) can inhibit CFTR function (Zaman et al., 2004; Marozkina et al., 2010). The molecular mechanisms underlying GSNO’s corrective effect on F508del-CFTR involve S-nitrosylation of specific chaperone proteins. First, S-nitrosylation of Hsp70/Hsp90 organizing protein (Hop/Stip-1) reduces its expression and inhibits its association with CFTR in the endoplasmic reticulum, facilitating F508del-CFTR maturation (Marozkina et al., 2010). Second, S-nitrosylation of Heat Shock Cognate 70 kDa protein (HSC70) at a critical cysteine in its ATP-binding domain enables the co-chaperone Csp to enhance F508del-CFTR folding and stability (Zaman et al., 2006). Third, GSNO interacts with E3 ubiquitin ligase C-terminus Hsc70 interacting protein (CHIP), inhibiting CHIP-CFTR interaction and reducing CFTR ubiquitination, which increases both mature and immature F508del-CFTR levels and enhances cell surface expression (Zaman et al., 2019). These mechanisms represent potential therapeutic targets for CF treatment through modulation of post-translational modifications in the CFTR maturation pathway (Zaman et al., 2019).

##### 5.1.7.3 Dysregulation of antioxidant defense systems

Proteomic analyses of CF cellular models and patient samples have revealed a complex dysregulation of antioxidant enzyme expression. CF epithelial models showed decreased expression of several crucial antioxidant enzymes including TRX-1, PRDX-1 and 6, CAT, and GST-pi (Chen et al., 2008). Similarly, nasal polyps from CF patients exhibited downregulation of PRDX-1, 2, and 6 compared to controls (Jeanson et al., 2014). Paradoxically, increased levels of mitochondrial SOD (MnSOD/SOD2) were observed in both CF bronchial epithelial cell models (Chen et al., 2008) and patient BALF samples (Kalsi et al., 2025). While SOD2 converts superoxide radicals to H_2_O_2_, the deficiency in downstream H_2_O_2_-detoxifying enzymes (CAT and peroxiredoxins) potentially leads to H_2_O_2_ accumulation, exacerbating oxidative stress (Chen et al., 2008). Glutathione peroxidases (GPxs), which utilize GSH for H_2_O_2_ detoxification, show no evident reduction in CF; in fact, elevated GPx levels have been reported in CF sputum (Dauletbaev et al., 2005) and increased extracellular GPx3 was identified in CF BALF (Kalsi et al., 2025). However, the effectiveness of these enzymes is likely limited by the characteristic GSH deficiency in CF, both in pulmonary epithelial lining fluid and systemically in plasma (Hewson et al., 2020). The elevated GPx in extracellular fluids may reflect release from inflammatory/damaged cells or a compensatory response compromised by GSH scarcity (Dauletbaev et al., 2004). Nrf2, the master regulator of antioxidant response, showed approximately 70% decreased expression and activity in CF cells compared to normal controls (Chen et al., 2008). This Nrf2 dysfunction explains the observed downregulation of multiple Nrf2-dependent antioxidant enzymes in proteomic studies. The consequences of Nrf2 impairment in CF include weakened intrinsic antioxidant defense capacity and increased intracellular H_2_O_2_ levels, which contribute to the overproduction of pro-inflammatory cytokines like IL-6 and IL-8, directly linking defective antioxidant response to CF’s characteristic chronic inflammation (Chen et al., 2008). Notably, experimental interventions with Nrf2-stabilizing compounds normalized H_2_O_2_ processing and significantly reduced inflammatory cytokine production in CF cells (Chen et al., 2008; Laselva et al., 2021).

##### 5.1.7.4 Oxidative stress and inflammatory pathways

The interplay between oxidative stress and inflammation is a defining characteristic of CF lung disease, creating a vicious cycle of damage. Proteomics has been instrumental in dissecting the molecular components of this interaction. Activated neutrophils release a potent arsenal of damaging agents, including ROS, various proteases (notably neutrophil elastase), and myeloperoxidase (MPO) (Pinzaru et al., 2023). MPO catalyzes the formation of hypochlorous acid (HOCl) and contributes to generating long-lived oxidants like chloramines, detected in high concentrations in CF sputum (Witko-Sarsat et al., 1995). Sputum proteomic studies consistently reveal high abundance of neutrophil-derived proteins, with levels of proteins like neutrophil elastase correlating inversely with lung function (FEV1) and positively with markers of inflammation and infection (Pattison et al., 2017). Mitochondria, primary sites of cellular energy production through oxidative phosphorylation, represent a major endogenous source of ROS and contribute significantly to CF pathophysiology (Velsor et al., 2006). Studies using CFTR-KO and CFTR-deficient human lung epithelial cell lines have demonstrated that dysfunctional CFTR is associated with significantly lower levels of mitochondrial GSH. This mitochondrial GSH depletion is accompanied by evidence of mitochondrial oxidative stress, including increased levels of 8-OHdG, and significant loss of aconitase activity, a mitochondrial enzyme particularly sensitive to inactivation by superoxide radical (Velsor et al., 2006).

##### 5.1.7.5 Impact of ETI on the oxidative stress proteome

Sputum proteomics has shown that ETI therapy induces a shift in the airway proteome; however, this new state is often described as “intermediate,” distinct from both the pre-treatment CF state and that of healthy controls, with evidence of incomplete resolution of neutrophilic inflammation (Maher et al., 2024). Similarly, plasma proteomics in pediatric CF patients after ETI initiation revealed only modest changes in some circulating inflammatory proteins, although key inflammatory pathways like NF-κB were affected (Ozuna et al., 2025). A consistent theme emerging from these and other studies is that despite the remarkable clinical improvements conferred by ETI, residual airway infection, oxidative stress, and inflammation often persist (Villella et al., 2025). This suggests that the pathological processes and tissue damage established over years of CFTR dysfunction may not be entirely reversible by the current levels of CFTR correction achieved, or that the restoration of CFTR function is insufficient to fully extinguish these self-perpetuating cycles of inflammation and oxidative damage.

The concept of an “intermediate state” post-ETI is pivotal (Maher et al., 2024): patients are significantly better, but their molecular profiles do not fully normalize to that of healthy individuals.

### 5.2 Lipidomics

In CF, oxidative stress and lipid dysregulation exhibit a bidirectional relationship, creating a detrimental feed-forward cycle. Elevated oxidative stress drives lipid peroxidation, generating reactive derivatives that further compromise cellular function, while disturbed lipid metabolism simultaneously enhances ROS production (Kleme and Levy, 2015; Drzymala-Czyz et al., 2024). Lipidomics, the comprehensive analysis of lipids within biological systems, offers crucial insights into this complex interplay, revealing altered metabolic pathways and potential therapeutic targets in CF pathophysiology (Ollero et al., 2011; Han and Gross, 2022). Unlike proteomic analyses, lipidomic investigations require specialized extraction protocols optimized for amphipathic molecules (Supplementary Table S4). The Bligh and Dyer method represents the predominant approach, employing chloroform-methanol solvent systems to effectively solubilize and recover diverse lipid classes across polarity ranges (Bligh and Dyer, 1959; Ollero et al., 2011). This fundamental distinction from protein extraction techniques reflects the unique physicochemical properties of lipids and their cellular compartmentalization. Various biological sources are utilized for these extractions, including CF cell models (IB3-1, CFBE41o-), clinical specimens (plasma, BALF, saliva), and animal models, each offering specific insights into CF lipid dysregulation. Mass spectrometry constitutes the cornerstone of modern lipidomic analysis in CF research. Liquid chromatography-mass spectrometry (LC-MS) separates lipids based on physicochemical properties prior to mass analysis, significantly enhancing detection of structurally similar species, including isobaric and isomeric molecules (Dei Cas et al., 2020). High-resolution mass spectrometry instruments utilizing Orbitrap or Time-of-Flight (TOF) technologies provide the mass accuracy essential for confident lipid identification. Alternative analytical approaches include Matrix-Assisted Laser Desorption/Ionization Time-of-Flight Mass Spectrometry (MALDI-TOF-MS), often coupled with thin layer chromatography (TLC-MALDI) for enhanced separation (Guerrera et al., 2009; Ollero et al., 2011). Direct Infusion Mass Spectrometry (shotgun lipidomics) offers high-throughput capabilities by introducing lipid extracts directly into the mass analyzer, though it requires exceptional mass resolution and/or tandem mass spectrometry (MS/MS) to differentiate similar lipid species effectively (Ryan and Reid, 2016; Supplementary Table S4).

#### 5.2.1 Results: lipidomics in CF

Comprehensive lipidomic investigations in CF have revealed multifaceted lipid dysregulation intimately connected with oxidative stress mechanisms. Significant phospholipid profile alterations characterize CF pathology, with diminished phosphatidylcholine and lysophosphatidylcholine species in patient’s plasma correlating with respiratory disease severity (Guerrera et al., 2009; Ollero et al., 2011). Recent advanced methodologies have identified specific oxidized phospholipid species (PC 40:5;O and PC 36:5;O), providing direct evidence of membrane oxidative modification (Zardini Buzatto et al., 2021). Concurrently, ceramide metabolism exhibits complex dysregulation, with apparent contradictions reflecting tissue-specific pathophysiology; some investigations report reduced ceramide concentrations in plasma (Guilbault et al., 2008; Wojewodka et al., 2011), while others demonstrate accumulation of long-chain ceramide species in pulmonary tissues (Veltman et al., 2021). Notably, elevated ratios of long-chain to very long-chain ceramide species in BALF correlate with inflammatory markers and lung disease severity, mechanistically linked to enhanced bacterial susceptibility and epithelial apoptosis (Zhang et al., 2008; Becker et al., 2010). Essential fatty acid imbalance constitutes another consistent aberration in CF, characterized by decreased linoleic acid and docosahexaenoic acid levels alongside relative arachidonic acid elevation, promoting pro-inflammatory eicosanoid production (Kleme and Levy, 2015; Drzymala-Czyz et al., 2024). Supporting this paradigm, elevated urinary 8-iso-prostaglandin F2α serves as a reliable biomarker of increased lipid peroxidation, correlating with pulmonary dysfunction (Ciabattoni et al., 2000; Veltman et al., 2021). Additional alterations manifest in ether-linked phospholipids, cholesterol esters, and glycosylated sphingolipids, with particular disruption of membrane cholesterol distribution crucial for CFTR protein trafficking (Dei Cas et al., 2020; Liessi et al., 2020; Cui et al., 2021). Therapeutic CFTR modulator therapies demonstrate promising effects on these lipid abnormalities. ETI enhances mutated CFTR protein expression while simultaneously remodeling sphingolipid composition (Dobi et al., 2025), increasing dihydrosphingolipids via modulation of Δ4-desaturase enzymes (Liessi et al., 2023; Ciobanu et al., 2024), and beneficially altering fatty acid metabolism (Veltman et al., 2021). Clinically, these interventions improve lipoprotein profiles, increasing anti-atherogenic HDL cholesterol while decreasing atherogenic LDL particles (Yuzyuk et al., 2023; Lonabaugh et al., 2024). Complementary antioxidant supplementation (vitamin E, GSH, β-carotene) effectively mitigates oxidative stress-induced lipid peroxidation, reducing isoprostane excretion and enhancing LDL oxidation resistance (Ciabattoni et al., 2000; Cantin et al., 2007; Veltman et al., 2021). These findings collectively establish the lipidomic profile as both a mechanistic framework and therapeutic target in CF, highlighting the critical intersection between lipid metabolism, oxidative stress and CFTR function in the disease pathophysiology.

### 5.3 Transcriptomics

As for proteomics and lipidomics, also transcriptomics has been applied in CF in the context of oxidative stress studies. Transcriptome is comprehensive of all the RNAs within a cell or a tissue, both the coding RNAs (mRNA) and the non-coding RNAs (i.e., long non-coding, small interfering RNAs) under certain conditions at a specific developmental stage (Casamassimi et al., 2017). Thus, it is possible to evaluate differences that intervene upon certain treatments, or in the presence of bacterial infections. Anyway, one should consider that different cells in the same tissue could also express different genes: single-cell analysis instead of bulk analysis could exclude bias given by the cellular composition from which RNA is isolated (Tang et al., 2009; Li and Wang, 2021).

#### 5.3.1 Transcriptomics methodologies

Two main approaches for transcriptomics are microarray and RNA-sequencing (RNA-Seq).

##### 5.3.1.1 Microarrays

Microarrays investigate a predefined set of genes, and the technique is based on the hybridization of RNA to specific cDNA sequences (named probes) immobilized on a solid support. These supports can contain thousands of gene probes distributed in a regular pattern of rows and columns to simplify the analysis. Briefly, high quality isolated RNAs are labelled with fluorescent dyes (i.e., cyanine-3 and -5) to cRNAs, that are purified and quantified. Then, sample cRNAs are hybridized to probes and fluorescent signal is measured. By using a dual colour labelling kit, it is possible to compare samples from two different conditions. The samples will compete for the probes and the ratio of the fluorescent signals measured at the two wavelengths will return a direct measurement of the relative abundance of the corresponding RNA (Galvin et al., 2004; Ramachandran et al., 2011; Casamassimi et al., 2017; Agapito and Arbitrio, 2022).

##### 5.3.1.2 RNA-seq

Conversely, RNA-sequencing is sequence-based and returns the quantification of all the transcripts with no bias on the genes probed, allowing also the detection of low-expressing genes that would not be identified with microarrays. Briefly, isolated RNAs are retrotranscribed to form a library of adapter-bound cDNA fragments. Adapters can be ligated on one or both sides of the fragments. Then, each molecule is sequenced from one end (single-end sequencing) or both ends (pair-end sequencing) with the high-throughput next-generation sequencing technology of choice. In general, single-end sequencing is sufficient in case of well-annotated organisms and frequently focuses only on mRNAs, while pair-end sequencing is recommended for *de novo* transcript discovery or isoform analysis. Optimal conditions for RNA-Seq depends on several factors such as the organism being studied and the aim of the research as explained elsewhere (Wang et al., 2009; Conesa et al., 2016; Li and Wang, 2021). Bulk RNA-Seq has been implemented to analyse single cell RNA (scRNA-Seq), to limit bias given by inter-cellular variability. This technology is based on the production of GEMs, microdroplets each containing a single cell derived from tissue homogenization, a retro transcription mix and a gel bead functionalized with oligo sequences. Each oligo sequence contains an adapter, a barcode to uniquely identify the cell source of RNA, a random tag for RNA identification and quantification, and an oligo-dT primer for mRNA binding. In this way, all the RNAs derived from the same cell possess the same barcode but are identified by unique tags. Barcoding allows multiplexing, the simultaneous scRNA-Seq of multiple samples, thus reducing the costs, increasing the throughput and improving the time to results (Cheng et al., 2021; Li and Wang, 2021).

#### 5.3.2 Transcriptomics applications in CF

Transcriptome profiling is a valuable tool in CF when studying the pathophysiology of the disease or when looking for new therapeutical targets (McKiernan et al., 2014; Kormann et al., 2017). Intriguingly, transcriptomic analysis has not been applied only to identify differences in gene expression between cells with or without a functional CFTR (Kamei et al., 2019; Declercq et al., 2021), but also to characterize the transcriptional activity of each cell type component of the lung epithelia (Zoso et al., 2019; Sun and Zhou, 2025) and to study pathogen virulence in CF patients (Worgall et al., 2005; Drevinek et al., 2008; Rossi et al., 2018; Irvine et al., 2019). Regulation of genes involved in the antioxidant response in CF have been identified in several studies, both with microarrays and RNA-Seq.

For example, Declercq and colleagues have performed transcriptomic analysis to study the effect on gene expression of CFTR depletion. In particular, they have used complementary models of CFTR silencing and blockade (by the CFTR inhibitor, CFTRinh-172) in freshly isolated human umbilical vein endothelial cells (HUVECs). Then, they have validated key findings *in vitro* and *in vivo* in *Cftr*-KO mice and *ex vivo* in CF patient-derived endothelial cells. Transcriptomics revealed that *CFTR* knock down cells are characterized by an upregulation of oxidative stress response-related genes like those expressing for GSH consuming enzymes (i.e., *GPX1*, *GPX4*), *SOD1* and *2*, peroxiredoxin and thioredoxins. These results were confirmed by flow cytometry. Moreover, LC-MS analysis followed to prove the depletion of GSH and NADPH, that confirmed the sustained oxidative stress. Further validation analyses were conducted with DCF and MitoSOX (Declercq et al., 2021). Similar results were obtained with a transcriptomic analysis following the pollution-caused CFTR decrease in 16HBE14o-cells. What has emerged is a pattern similar to that observed in CF: a diminished amount of CFTR leads to an upregulation of *SOD1* and a downregulation of *CAT*. These events together induce the accumulation of H_2_O_2_ within the cells. Moreover, an upregulation of *HMOX1* favours the accumulation of Fe^2+^ that can react with H_2_O_2_ in a Fenton-like reaction thus producing the highly reactive ROS hydroxyl radical. This worsens the oxidative stress and finally leads to DNA damages (Stermann et al., 2022).

A different application of transcriptomics in CF consists in the study of gene regulation in pathogens typically infecting CF patients, for the identification of mechanisms involved in their adaptation and virulence. For example, transcriptomic analysis of a strain of *P. aeruginosa*, isolated from a mouse model of chronic infection, but originally from a CF patient, was performed to identify transcriptional modifications responsible for the phenotypic change from mucoid to small colonies vescicles (SCV). The latter phenotype is typical of lung adaptation and loss of functionality in CF patients. Authors have performed RNA-seq and discovered that, among all the genes regulated in the phenotypic change, half of those upregulated are involved in the response to oxidative stress (Irvine et al., 2019). Analogously, another important pathogen in CF, the *B. cenocepacia*, isolated from CF sputum have activated genes that confer protection against ROS, as demonstrated with microarray-based studies (Drevinek et al., 2008). As demonstrated by the previous examples, transcriptional analysis can help in the identification of virulence genes of pathogens that represent the principal cause of mortality in CF patients. Resistance to oxidative stress in these pathogens help them survive the attack of neutrophils. The identification of these genes is a step forward to a possible therapy in case of antibiotic resistance. When performing transcriptomics, it is important to validate the results with studies of protein amount or enzyme activity, because of regulatory mechanism that could intervene after transcription.

## 6 Nonenzymatic antioxidants

### 6.1 GSH

GSH is a tripeptide constituted by glycine, glutamate and cysteine and its intracellular amount is an indicator of the redox state of the cells. When oxidised, it forms the disulfide GSSG that can be reduced back to GSH by the glutathione reductase (GR). Methods for GSH quantification can be categorized into spectrophotometric-based and separative methods. The latter are based on the use of HPLC (Giustarini et al., 2003; Hector et al., 2010; Hou et al., 2018), gas chromatography (Bollenbach and Tsikas, 2022) or capillary electrophoresis (Muscari et al., 1998) and sometimes they require the use of electrochemical or mass spectrometer detectors to increase the sensitivity (Cappiello et al., 2013). The most used spectrophotometric-based method for GSH quantification is the GSH recycling assay. It is based on the GR recycling ability to regenerate GSH from GSSG. Specifically, this assay involves the oxidation of GSH by the Ellman’s reagent 5,5′-dithio-bis(2-nitrobenzoic acid) (DTNB) to form the yellow derivative 5′-thio-2-nitrobenzoic acid (TNB), whose absorbance is measured at 412 nm. This reaction generates also the oxidized glutathione-TNB (GS-TNB) adduct that is then reduced back to GSH by GR consuming NADPH and liberating another TNB, thus amplifying the signal (Tietze, 1969). Thanks to the presence of GR, a single molecule of GSSG is reduced to two molecules of GSH, so that the presented method measures total GSH as the sum of reduced and oxidized GSH ([GSH]_total_ = [GSH] + 2 x [GSSG]) (Rahman et al., 2007). A few years later than Tietze′s assay development, Griffith and colleagues modified it to make possible the parallel quantification of the oxidized fraction GSSG. In fact, since the ratio GSH:GSSG is only 1:10 and considered that GSH oxidation is favored, GSSG quantification was considered a difficult procedure. Sulphydryl group derivatization with 2-vinylpyridine masks the reduced GSH so that only the GSH derived from the GR-mediated reduction of GSSG can react with DTNB. In this way the measured absorbance depends only on GSSG, ignoring the reduced fraction originally in the sample (Griffith, 1980).

### 6.2 Vitamins

In the clinical management of CF, accurate assessment of vitamin status is critical due to the high prevalence of malabsorption, particularly of fat-soluble vitamins (A, D, E, K), stemming from pancreatic insufficiency. The primary matrix for determining systemic vitamin concentrations in CF patients is serum or plasma. Advanced analytical techniques, predominantly chromatographic methods, are employed for this purpose. LC-MS/MS is widely regarded as a gold standard due to its inherent sensitivity, specificity, and capability for simultaneous quantification of multiple vitamins and their metabolites from limited sample volumes, which is particularly advantageous in pediatric CF patients (Konieczna et al., 2016; Juhasz et al., 2021). Sample preparation for LC-MS/MS typically involves steps such as protein precipitation or liquid-liquid extraction to isolate the target vitamins from the complex biological matrix before chromatographic separation and subsequent mass spectrometric detection (Konieczna et al., 2016; Ma et al., 2024). HPLC coupled with detectors like UV or fluorescence is also a well-established and reliable method for quantifying specific vitamins in CF, utilizing similar extraction procedures prior to chromatographic separation and detection (Francalanci et al., 2023). While chromatographic methods offer comprehensive profiling, ELISA have also found application in vitamin determination in CF, albeit more commonly for specific vitamins such as vitamin D (Belle et al., 1995; Jagannath et al., 2020).

### 6.3 Assessment of total antioxidant capacity (TAC)

CF pathophysiology involves disrupted cellular equilibrium alongside persistent inflammatory responses that drive progressive tissue deterioration, with particular severity in pulmonary manifestations (O’Sullivan and Freedman, 2009). This pathological state creates an imbalance where ROS and RNS production exceeds the neutralizing ability of intrinsic antioxidant systems, thereby establishing conditions of oxidative stress (Rahman and MacNee, 2000). The measurement of TAC represents a comprehensive evaluation methodology that quantifies the collective ability of biological systems to neutralize oxidative damage, offering valuable understanding of redox dysregulation and guiding therapeutic interventions focused on enhancing cellular protective mechanisms (Cao and Prior, 1999).

Multiple analytical techniques utilizing spectrophotometric and fluorometric detection have emerged for TAC quantification in biological specimens, with each methodology employing unique chemical mechanisms to evaluate integrated antioxidative responses. The principal methods for TAC determination encompass ABTS, FRAP, DPPH, BAP, and TRAP analytical procedures.

#### 6.3.1 ABTS (2,2′-azinobis-(3-ethylbenzothiazoline-6-sulfonic acid)) methodology

The ABTS protocol evaluates antioxidant efficacy through the neutralization of pre-generated ABTS·^+^ radical cations (Deadman, 1973). These chromogenic radical species, produced via ABTS oxidation using potassium persulfate or comparable oxidizing agents, demonstrate pronounced light absorption across the visible range, particularly at 734 nm wavelength. Sample antioxidants interact with ABTS·^+^ through either single electron transfer (SET) or hydrogen atom transfer (HAT) pathways, leading to radical quenching and a proportional reduction in measured absorbance (Deadman, 1973). Quantification typically employs Trolox (water-soluble vitamin E derivative) as reference standard, with results expressed as Trolox Equivalent Antioxidant Capacity (TEAC) units. This analytical approach demonstrates broad applicability across both water-soluble and fat-soluble antioxidant compounds (Deadman, 1973).

#### 6.3.2 FRAP (ferric reducing antioxidant power) methodology

FRAP analysis determines sample reducing capacity by measuring electron donation potential (Benzie and Strain, 1996). Within acidic environments (pH 3.6), antioxidant molecules facilitate the reduction of ferric-2,4,6-Tris(2-pyridyl)-s-triazine (Fe^3+^-TPTZ) complexes to their ferrous counterparts (Fe^2+^-TPTZ). This chemical transformation produces a distinctive blue chromophore (Benzie and Strain, 1996). Spectrophotometric monitoring occurs at 593 nm, where absorbance increases correlate directly with reducing antioxidant concentrations. Quantification employs either ferrous ion equivalents or Trolox standardization. Notably, FRAP methodology predominantly captures electron-transfer mechanisms while potentially underestimating hydrogen atom transfer-based radical scavenging processes (Benzie and Strain, 1996).

#### 6.3.3 DPPH (2,2-diphenyl-1-picrylhydrazyl) methodology

DPPH analysis represents an established approach for evaluating antioxidant radical-neutralizing capabilities (Brand-Williams et al., 1995). The method employs stable DPPH· synthetic radicals, characterized by purple coloration and maximum absorption at 517 nm. Antioxidant exposure leads to DPPH· reduction to its non-radical state (DPPH-H) through hydrogen atom acceptance (HAT mechanism) or electron acquisition (SET mechanism) (Brand-Williams et al., 1995). This transformation results in decreased 517 nm absorbance, monitored either kinetically or at predetermined endpoints. Data presentation includes percentage radical scavenging calculations or standardization against reference antioxidants such as ascorbic acid or Trolox (Brand-Williams et al., 1995). This methodology proves particularly useful for rapid *in vitro* screening of free radical interception capacity.

#### 6.3.4 BAP (biological antioxidant potential) methodology

BAP testing utilizes commercially available assay systems frequently implemented in clinical diagnostic environments for evaluating collective reducing potential in biological matrices including serum and plasma (Celi et al., 2010). The procedure involves sample-mediated reduction of proprietary ferric-chromogen complexes, generating colored ferrous products (Celi et al., 2010). Spectrophotometric quantification (wavelengths typically 505–520 nm depending on kit specifications) measures color intensity proportional to sample reducing capacity. Standardization against predetermined references (commonly ferrous ion-based) provides rapid systemic antioxidant potential assessment (Celi et al., 2010). While offering high-throughput convenience for clinical laboratories, BAP analysis provides composite measurements without identifying individual antioxidant contributors.

#### 6.3.5 TRAP (total radical-scavenging antioxidant parameter) methodology

TRAP analysis specifically quantifies sample effectiveness in neutralizing peroxyl radicals (R-OO·), offering critical information regarding cellular protection against lipid peroxidation processes (Uotila et al., 1994). Systematic peroxyl radical production occurs via controlled thermal breakdown of radical initiators, particularly 2,2′-Azobis(2-amidinopropane) dihydrochloride (ABAP) (Uotila et al., 1994). The methodology tracks oxidative damage inflicted upon target molecules or detection probes. When employing fluorescent probes such as R-phycoerythrin, the analysis measures fluorescence signal deterioration resulting from peroxyl radical-induced oxidation. This fluorescence monitoring typically utilizes excitation wavelengths spanning 488–565 nm with emission detection ranging 575–675 nm (commonly centered around 580 nm). Alternatively, when chemiluminescent probes are utilized, direct measurement of light emission decay provides the analytical signal (Uotila et al., 1994).

### 6.4 Results: non-enzymatic antioxidants

#### 6.4.1 GSH

Dysregulation of the key antioxidant GSH is a widely investigated hallmark of CF. In peripheral blood, CF patients consistently exhibit altered GSH status: reduced intracellular levels in neutrophils and lymphocytes (Lands et al., 1999; Tirouvanziam et al., 2006), the latter correlating with lung function, and diminished plasma GSH and GSH:GSSG ratios (Roum et al., 1993; Lagrange-Puget et al., 2004; Innis et al., 2007). Despite persistent oxidative stress markers (Olveira et al., 2013), interventions like N-acetylcysteine or specific supplements show some restorative promise (Tirouvanziam et al., 2006; Innis et al., 2007). The airway presents a more complex GSH profile; while BALF from CF patients and CFTR-deficient models typically shows depleted GSH, exacerbated by infection (Gould et al., 2010; Kettle et al., 2014; Dickerhof et al., 2017), sputum studies have surprisingly reported higher total GSH (Dauletbaev et al., 2004). Furthermore, one murine study found no GSH:GSSG alterations in lung tissue or BALF (Velsor et al., 2001), highlighting compartmental intricacies. Nevertheless, aerosolized GSH (Roum et al., 1999) and hypertonic saline (Gould et al., 2010) can augment airway GSH. Cellular investigations reveal further nuances. CFTR deficiency is linked to markedly lower mitochondrial GSH (Velsor et al., 2006) and often reduced intracellular GSH in epithelial cells, which can be partially restored by CFTR modulators like lumacaftor (de Bari et al., 2018). However, contrasting findings of normal or even slightly higher intracellular GSH in certain CF cell lines exist (Bergamini et al., 2009). Promisingly, compounds such as γ-glutamylcysteine and whey protein hydrolysates can effectively increase intracellular GSH in CF bronchial epithelial cells (Grey et al, 2003; Vilela et al., 2006; Hewson et al., 2020).

#### 6.4.2 Vitamins

Altered vitamin status, linked to malabsorption and pulmonary disease severity, is a well-documented challenge in CF (Wysocka-Wojakiewicz et al., 2022). Deficiencies in fat-soluble vitamins (A, D, E, K) are particularly prevalent. Consistently low plasma vitamin A and E necessitate supplementation (Brown and Kelly, 1994; Portal B. C. et al., 1995; Benabdeslam et al., 1999; Lai et al., 2022), while highly prevalent vitamin D deficiency, impacting bone and potentially lung health, faces repletion challenges despite supplementation efforts (Lai et al., 2022; Francalanci et al., 2023; Farahbakhsh et al., 2024). Impaired vitamin K status, especially with pancreatic insufficiency, also risks coagulopathy and compromised bone mineralization (Krzyzanowska et al., 2015). Notably, CFTR modulator therapies variably affect these vitamins: lumacaftor/ivacaftor increased plasma vitamin A but moderately decreased the vitamin E/cholesterol ratio (Sommerburg et al., 2021), whereas ETI boosted vitamins A and D, leaving levels of E and K reportedly stable (Hergenroeder et al., 2023).

Though less universally deficient, water-soluble vitamins can be suboptimal and play distinct roles in CF. Vitamin C, for instance, may activate defective CFTR *in vitro* (Fischer et al., 2004), and its supplementation can positively influence vitamin E levels, suggesting oxidative stress mitigation (Traber et al., 2022). Among B vitamins, while 5-methyltetrahydrofolate (5-MTHF) and vitamin B12 supplementation showed potential cellular benefits (Scambi et al., 2009), paradoxically high, often supra-physiological, serum B12 levels are frequently observed in highly supplemented children with pancreatic insufficiency (Maqbool et al., 2014). Early studies indicated generally adequate riboflavin (B2) and pyridoxine (B6) levels in treated patients (Congden et al., 1981). However, low plasma concentrations of B6’s active form, pyridoxal 5′-phosphate, are a common abnormality (Faraj et al., 1986). The metabolic importance of folate (Scambi et al., 2009) is also recognized, and rare isolated vitamin B2 deficiencies have been documented in specific clinical contexts (McCabe, 2001).

#### 6.4.3 TAC

Few studies have employed TAC methodologies to highlight alterations in antioxidant capacity in CF. Notably, Langley and colleagues reported that despite elevated plasma concentrations of individual antioxidants like ascorbic acid, uric acid, and sulfhydryl groups compared to healthy controls, the CF patients exhibited a significantly reduced TRAP (Langley et al., 1993). Furthermore, they observed a strong negative correlation between TRAP values and high plasma ascorbic acid levels in CF patients, suggesting that high concentrations of ascorbic acid might exert a pro-oxidant effect in the context of CF, thereby diminishing the overall extracellular antioxidant defense (Langley et al., 1993).

Expanding on these observations, serum total antioxidant capacity using ABTS and FRAP assays in a cohort of CF patients was studied (Galiniak et al., 2022). Results indicated a significantly lower overall serum TAC by the FRAP assay in CF patients compared to controls, although the ABTS assay did not show this overall difference (Galiniak et al., 2022). This study further demonstrated that reduced TAC was associated with increased CF disease severity. However, TAC levels did not differ significantly among CF patients based on the presence or type of bacterial infection (Galiniak et al., 2022). The Galiniak et al. study also found that TAC measured by ABTS correlated negatively with age and positively with FEV1.

## 7 Antioxidant enzyme activity and levels

The assessment of antioxidant enzyme activity in CF is crucial for understanding the redox imbalances that contribute to disease pathogenesis. This section details the common methodologies employed for measuring SOD, CAT, and GPx activity in biological samples relevant to CF, summarized in Supplementary Table S5.

### 7.1 SOD assays

**FIGURE 2:**
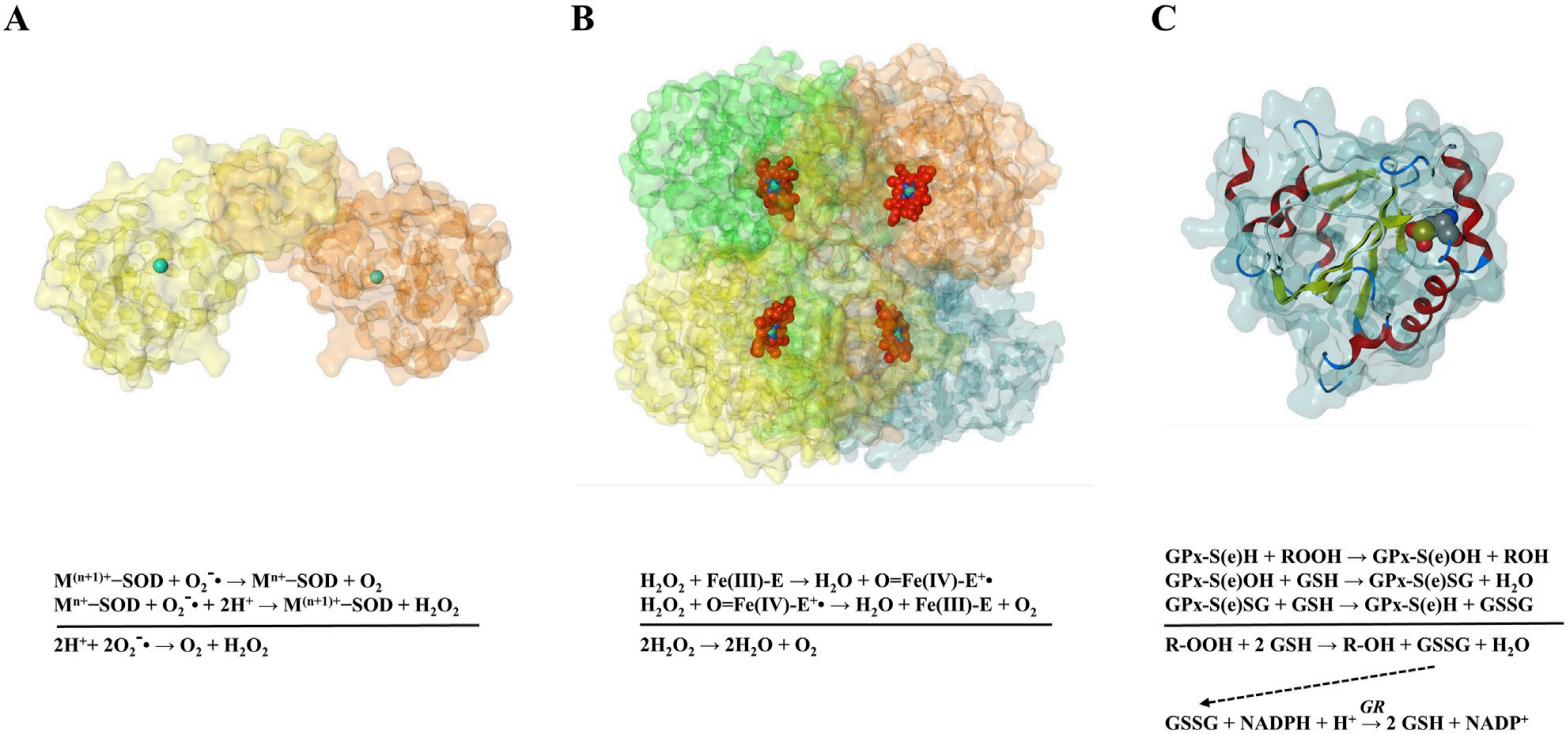
**(A)** Catalytic mechanism of SOD. In this initial step, the oxidized form of the metal cofactor (M^(n+1)+^) within the SOD enzyme reacts with a superoxide radical. The metal ion is reduced to a lower oxidation state (M^n+^), while the superoxide radical is oxidized to molecular oxygen (O_2_). Subsequently, the reduced form of the metal cofactor (M^n+^) in SOD reacts with a second superoxide radical and two protons (2H^+^). This results in the re-oxidation of the metal ion back to its initial higher oxidation state (M^(n+1)+^), and the superoxide radical is reduced to hydrogen peroxide (H_2_O_2_). **(B)** Catalytic mechanism of CAT. The resting ferric state (Fe(III)) of the enzyme (E) reacts with one molecule of H_2_O_2_. This leads to the formation of an oxoiron(IV) and the release of one water molecule. Oxoiron(IV) then reacts with a second molecule of H_2_O_2_. This reaction results in the reduction of the oxoiron(IV) back to the enzyme’s resting ferric state (Fe(III)−E), the release of O_2_ and the formation of another water molecule. **(C)** Catalytic mechanism of Glutathione Peroxidase (GPx). Active GPx, contains a cysteine (S) or selenocysteine (Se) residue in its reduced sulfhydryl or selenol state (GPx−S(e)H). This sulfhydryl/selenol reacts with a hydroperoxide (R-OOH, where R can be an organic group or hydrogen), which is reduced to the corresponding alcohol (R-OH) or a water molecule. The S(e) atom in GPx is oxidized to the sulfenic or selenenic state (GPx−S(e)OH). The sulfenic/selenenic intermediate then reacts with a molecule of reduced glutathione (GSH). This reaction forms a mixed sulfenyl/selenenyl sulfide adduct between the enzyme and glutathione (GPx−S(e)SG), and a water molecule is released. The GPx−S(e)SG intermediate subsequently reacts with a second molecule of GSH. This step regenerates the active, reduced form of the enzyme (GPx−S(e)H) and produces oxidized glutathione (GSSG). GSH must be regenerated from GSSG. This crucial step is catalyzed by the enzyme Glutathione Reductase (GR). GR utilizes nicotinamide adenine dinucleotide phosphate (NADPH) as a reducing agent, transferring electrons to GSSG to regenerate two molecules of GSH. Figure generated using Molecular Operating Environment (MOE), version 2024.06.01.

SODs are metalloenzymes that catalyze the dismutation of the superoxide anion (O_2_^−^·) into molecular oxygen (O_2_) and H_2_O_2_, forming a primary line of defense against oxidative stress (McCord and Fridovich, 1969; Figure 2A). Spectrophotometric methodologies are extensively utilized for quantifying SOD activity, primarily based on the principle that SOD catalytically inhibits reactions driven by superoxide radicals. These indirect assays precisely measure SOD activity by assessing the attenuation in the rate of a chromogenic reaction critically dependent on superoxide presence. A foundational technique involves monitoring the SOD-mediated inhibition of ferricytochrome c reduction by superoxide, typically observed as a change in absorbance at 550 nm, already detailed for superoxide anion quantification (McCord and Fridovich, 1969; Beauchamp and Fridovich, 1971). Another prevalent approach hinges on the reduction of nitroblue tetrazolium (NBT) by superoxide, yielding a discernible blue formazan product; SOD competes effectively for superoxide, thereby diminishing formazan formation, which is detected as a decrease in absorbance, generally between 540 nm and 570 nm (Beauchamp and Fridovich, 1971; Misra and Fridovich, 1972; Winterbourn et al., 1975). Furthermore, the superoxide-catalyzed autooxidation of specific compounds forms the basis of alternative spectrophotometric assays. The autooxidation of pyrogallol under alkaline conditions generates superoxide, and the SOD-dependent inhibition of this process, tracked by a reduction in the rate of absorbance increase (at 420 nm or 440 nm), constitutes a well-established assay (Marklund and Marklund, 1974). Similarly, the autooxidation of epinephrine at alkaline pH to produce adrenochrome is a superoxide-mediated event; SOD inhibits adrenochrome formation, which is typically followed by monitoring the decrease in absorbance around 480 nm (Misra and Fridovich, 1972), with a refined version of this assay utilizing changes at 320 nm (Sun and Zigman, 1978).

### 7.2 CAT assays

CAT is a heme-containing enzyme that catalyzes H_2_O_2_ decomposition into water and O_2_, providing cellular protection against oxidative damage (Aebi, 1984; Figure 2B). The classical spectrophotometric method monitors H_2_O_2_ UV absorption decrease at 240 nm during CAT-mediated degradation. In this approach, CAT-containing samples are introduced to defined H_2_O_2_ concentrations in phosphate buffer (pH 7.0), with absorbance decline rates proportional to enzymatic activity. Despite its simplicity, this method faces interference from co-absorbing compounds at 240 nm (Aebi, 1984). Alternative indirect spectrophotometric methods quantify residual H_2_O_2_ after defined incubation periods by generating colorimetric products inversely correlated with CAT activity. One such method couples pHPA with HRP, where remaining H_2_O_2_ serves as HRP substrate, catalyzing pHPA oxidation to produce measurable signals (Guilbault et al., 1968; Pick and Keisari, 1980). A more recent approach utilizes hydroquinone, anilinium sulfate, and ammonium molybdate reactions with unconsumed H_2_O_2_, forming quantifiable purple compounds (Hadwan and Ali, 2018). While offering greater sensitivity and visible-range measurements with reduced interference, these endpoint assays preclude continuous monitoring and require precise reaction termination timing. In-gel activity assays (zymography) provide complementary analysis following native polyacrylamide gel electrophoresis. After H_2_O_2_ incubation, gels are stained with ferric chloride and potassium ferricyanide, forming Prussian blue precipitates except in regions of CAT activity, which appear as clear bands against stained backgrounds (Pezzoni et al., 2018). This technique facilitates isoform identification based on electrophoretic mobility. Physiologically, erythrocytes exhibit high CAT activity for hemoglobin protection, typically normalized to hemoglobin content (Vitai and Goth, 1997). CAT activity is normally low in plasma or serum; elevated extracellular levels may indicate hemolysis or tissue injury causing enzyme leakage (Goth, 1991).

### 7.3 GPx assays

GPx represents a critical enzyme family in cellular antioxidant defense, catalyzing the reduction of H_2_O_2_ and organic hydroperoxides (R-OOH) to water and corresponding alcohols using reduced GSH as electron donor. Most mammalian GPx isoforms (e.g., GPx1, GPx3, GPx4) are selenoenzymes, containing selenocysteine at their catalytic center. GPx activity quantification predominantly employs the coupled enzyme spectrophotometric assay developed by Paglia and Valentine (Paglia and Valentine, 1967) and subsequently refined (Maiorino et al., 1988; Liu et al., 2022). This indirect method links the primary GPx reaction to a secondary enzymatic reaction with measurable spectrophotometric properties. Initially, GPx reduces hydroperoxides (H_2_O_2_, cumene hydroperoxide, or tert-butyl hydroperoxide) using GSH. The resulting oxidized GSSG is subsequently reduced by GR back to GSH, with NADPH as the reducing equivalent source (Figure 2C). The assay monitors NADPH oxidation to NADP^+^ via absorbance decrease at 340 nm. By maintaining excess GR, hydroperoxide, GSH, and NADPH, the reaction rate becomes solely dependent on GPx activity, enabling continuous, real-time kinetic measurement (Figure 2C). Alternative direct assays quantify GSH consumption over defined incubation periods with hydroperoxide substrates, calculating GPx activity from initial and final GSH concentrations (Razygraev et al., 2018). Zymography visualizes GPx isoenzyme activity after gel electrophoresis separation. These techniques involve incubating electrophoresed gels with GPx substrates and detecting GSH-depleted regions. Typically, tetrazolium salts are reduced to colored formazan products in GSH-containing areas, while GPx-active zones appear as clear bands against colored backgrounds (Lin et al., 2002). Biologically, GPx distribution varies across compartments: erythrocytes exhibit significant GPx1 activity for hemoglobin and membrane protection (Ravn-Haren et al., 2006); plasma contains kidney-synthesized GPx3 for extracellular defense, influenced by selenium availability (Ottaviano et al., 2009); and airway secretions utilize GPx for local hydroperoxide detoxification. The coupled spectrophotometric assay faces potential interference from factors affecting GR, non-specific NADPH consumption, or GSH degradation, potentially compromising measurement accuracy.

### 7.4 Results: antioxidant enzyme activities and levels in CF

The chronic inflammation and recurrent infections inherent to CF create a state of heightened oxidative stress, leading to investigations into the status of the antioxidant enzyme defense system. This section reviews findings on SOD, CAT, and GPx activities in various biological compartments of CF patients compared to controls.

#### 7.4.1 SOD

Erythrocyte lysates commonly assess intracellular SOD activity (predominantly Cu/Zn-SOD/SOD1), normalized to hemoglobin content for comparability (Matkovics et al., 1982). Plasma or serum measures extracellular SOD (EC-SOD/SOD3), though plasma may contain SOD1 released from lysed cells, affecting interpretation (Laskowska-Klita and Chelchowska, 2001; Olveira et al., 2013). Sputum and BALF provide direct insight into airway antioxidant defenses, with sputum typically processed into a soluble phase (“sol”) for enzyme analysis. However, sputum’s inherent complexity (mucus, cells, bacteria, mediators) challenges assay standardization, necessitating meticulous sample processing to preserve enzyme activity and minimize interference (Dosanjh, 2008). SOD activity in CF exhibits varied, compartment-specific alterations reflecting complex redox imbalance. Initial research demonstrated increased SOD activity in erythrocytes of CF children and heterozygous parents compared to controls, potentially representing an adaptive response to systemic oxidative stress (Laskowska-Klita and Chelchowska, 2001). Conversely, more recent studies in bronchiectasis patients, including CF cohorts, reported decreased plasma SOD activity, potentially from increased consumption due to heightened extracellular superoxide, reduced synthesis, or impaired EC-SOD release (Olveira et al., 2013). Animal models support tissue-specific changes, with decreased pancreatic Cu/Zn-SOD activity observed in CF porcine models (O’Malley et al., 2022). These varying observations highlight the critical distinction between enzyme concentration and actual catalytic activity, as evidenced in other inflammatory conditions where plasma SOD1 concentration increased while total activity remained unchanged (Sciskalska et al., 2020). Within airway secretions, CF sputum sol attenuates superoxide radical production by phorbol-stimulated control neutrophils in a time- and concentration-dependent, heat-labile manner, suggesting inhibitory enzymatic factors (Dosanjh, 2008). This effect is not mere scavenging; its inhibition by exogenous SOD indicates CF sol interferes with superoxide generation or availability. Paradoxically, while reducing superoxide, CF sputum can impair neutrophil bactericidal capacity (Fantone et al., 2021; 2023), partly linked to reduced phagosomal ROS production, essential for bacterial clearance. Investigations using CF cell lines reveal intrinsic SOD alterations linked to CFTR dysfunction. Pancreatic (CFPAC-1) and tracheal (CFT-2) CF cell lines exhibited reduced Mn-SOD and Cu/Zn-SOD expression compared to controls (Rottner et al., 2011). Despite similar EC-SOD expression, its enzymatic activity was significantly reduced in CF cells. Treatment with SOD mimetic (MnTMPyP) reduced the increased apoptosis sensitivity of CF cells, implicating superoxide-mediated oxidative stress (Rottner et al., 2011). These findings collectively highlight that impaired SOD function in CF can result from reduced expression or diminished catalytic activity, underscoring the necessity of functional assays beyond mere protein detection.

#### 7.4.2 CAT

In the context of CF, the presence and activity of CAT in airway secretions like sputum and BALF are significant for local H_2_O_2_ detoxification. Studies have quantified both CAT concentration and activity in CF sputum supernatants (Worlitzsch et al., 1998). Furthermore, tissue and cell lysates, particularly from bronchiolar epithelium which is a major site of CAT expression in healthy lungs, are highly relevant for investigating CAT’s contribution to lung pathology, including that observed in CF (Odajima et al., 2010). The status of CAT activity in CF patient blood has been investigated, yielding conflicting reports. Older studies indicated increased erythrocyte CAT activity in children with CF and their heterozygous parents compared to controls (Matkovics et al., 1982). Similarly, a study in adults with bronchiectasis, including a CF subgroup, reported increased plasma CAT (Olveira et al., 2013), potentially reflecting compensatory upregulation against chronic systemic oxidative stress (Galli et al., 2012). Conversely, a more recent study in CF children found significantly lower CAT activity after confounder adjustment (Laskowska-Klita and Chelchowska, 2001). These discrepancies likely stem from differences in patient populations, methodologies, or the specific blood compartment analyzed (erythrocytes vs. plasma), noting that plasma CAT is usually low, with increases potentially indicating cell leakage during inflammation (de Camargo et al., 2021). CF airways feature intense inflammation and high H_2_O_2_ production from phagocytes (Galli et al., 2012). CAT protein and activity are detectable in CF sputum, with high levels reported in some studies (Worlitzsch et al., 1998; Dauletbaev et al., 2005), contributing significantly to local H_2_O_2_ detoxification within the lumen (Worlitzsch et al., 1998). Despite high oxidant production, exhaled breath condensate H_2_O_2_ levels in CF patients are often similar to controls (Worlitzsch et al., 1998; Ho et al., 1999). This paradox is likely due to efficient H_2_O_2_ scavenging by CAT and other antioxidants in airway lining fluid and sputum (Worlitzsch et al., 1998; Mishra and Imlay, 2012). CF sputum extracts can neutralize H_2_O_2_ and protect cells *in vitro*, a protective effect sustained even after CAT inactivation by inhibitors (Dauletbaev et al., 2005). This highlights the crucial role of other antioxidant systems, particularly GPxs and reduced thiols, in CF sputum H_2_O_2_ neutralization (Dauletbaev et al., 2005). Conversely, viral infections like RSV decrease lung Cat in mice (Hosakote et al., 2011; Ansar et al., 2020), while chronic bacterial infection, specifically *P. aeruginosa* colonization, is associated with lower systemic CAT in CF children (Bennemann et al., 2022), suggesting infections can compromise CAT activity systemically or locally. Tissue-specific CAT status in CF also varies. The CF porcine pancreas showed no significant difference in CAT activity compared to non-CF animals (O’Malley et al., 2022). This contrasts with lung fibrosis models, which share some features with chronic CF lung damage, where decreased CAT activity, mRNA, and protein are observed, notably in bronchiolar epithelium (Odajima et al., 2010; Betsuyaku et al., 2013). Studies in acatalasemic mice demonstrated increased susceptibility to bleomycin-induced lung injury and fibrosis, highlighting CAT’s protective role in mitigating fibrotic lung disorders (Odajima et al., 2010).

#### 7.4.3 GPx

Studies highlight GPx’s substantial role, with abundant reduced thiols, in detoxifying H_2_O_2_ in CF sputum, particularly when CAT is inhibited or overwhelmed (Dauletbaev et al., 2004). Assessing GPx activity in tissue homogenates (e.g., lung, pancreas) or cell lysates (e.g., airway epithelial cells) is vital for understanding its contribution to CF pathology in affected organs. Glutathione peroxidase (GPx) activity in the blood of CF patients has shown some variability across studies. Older findings reported normal whole blood GPx in infants and children with CF despite lower whole blood selenium levels, suggesting potential compensatory mechanisms or non-critically limiting selenium (Ward et al., 1984). In contrast, a more recent study in CF children found significantly lower systemic GPx activity after adjusting for confounders (Bennemann et al., 2022). Given the common pancreatic insufficiency in CF leading to selenium malabsorption, reduced GPx activity is plausible (Winklhofer-Roob et al., 1998; Baker, 2008; Zheng and Mostamand, 2023), particularly for plasma GPx (GPx3), which is closely linked to selenium availability (Ottaviano et al., 2009). The glutathione system (GSH and GPx) is a major pulmonary antioxidant defense. Studies on CF sputum report surprisingly high levels of total GSH (predominantly reduced GSH) and GPx activity (Dauletbaev et al., 2004). Median total GSH in CF sputum was significantly higher than in non-CF controls, with over 90% as GSH in CF compared to <50% in controls (Dauletbaev et al., 2004). This abundance contributes to sputum’s H_2_O_2_-detoxifying capacity, sustained even after CAT inactivation, highlighting the GPx system’s importance in airway secretions (Dauletbaev et al., 2005). These high sputum GSH levels starkly contrast with reports of low GSH in BALF from the lower airways of CF children (Kettle et al., 2014; Dickerhof et al., 2017). This suggests compartmentalization: while expectorated sputum may be GSH-rich, the epithelial lining fluid of smaller, distal airways appears deficient (Gao et al., 1999; Kettle et al., 2014). This deficiency is likely linked to CFTR dysfunction affecting apical GSH transport (Kariya et al., 2007; Kettle et al., 2014). Viral infections, like RSV, also decrease lung GPx activity in mice (BALF) (Hosakote et al., 2011; Ansar et al., 2020). Efforts to augment airway GSH via inhalation have been explored. However, a large randomized controlled trial of inhaled GSH in CF patients increased sputum GSH but yielded no clinically relevant improvements in lung function, exacerbations, or oxidative stress markers (Griese et al., 2013). This outcome suggests that delivering GSH to bulk sputum may not correct the underlying oxidative imbalance or restore GPx-mediated protection effectively, possibly because GSH does not reach the critical distal airway site, local GPx activity is limited (e.g., by selenium), or the oxidant burden is overwhelming. Direct data on GPx activity in CF cellular or specific tissue models (beyond blood/airway secretions) are limited. A study on the CF porcine model found no significant difference in pancreatic GPx activity between CF and non-CF animals (O’Malley et al., 2022). However, research using CF airway epithelial cell lines (e.g., IB3-1) has shown decreased GPx4 levels under specific externally induced pro-oxidative stress (Maniam et al., 2021).

#### 7.4.4 Other enzymes involved in redox homeostasis and metabolism in CF

Beyond the well-characterized antioxidant enzymes such as SOD, GPx, and CAT, variations in other enzymatic activities also contribute to the complex redox imbalance and metabolic dysfunction observed in CF. GR, crucial for maintaining the pool of reduced GSH, has shown varied alterations depending on the cell type or tissue examined; studies have reported GR activity to be unvaried in erythrocytes of CF children (Carmagnol et al., 1983), while intracellular GR activity was significantly higher in lung tissue from *Cftr*-KO mice (Velsor et al., 2001), and conversely, significantly decreased in CF bronchial epithelial cells compared to WT controls (de Bari et al., 2018). Another enzyme involved in GSH conjugation and detoxification, Glutathione S-Transferase (GST), was found to be significantly increased in erythrocytes from children with CF (Carmagnol et al., 1983). In the context of mitochondrial function and its susceptibility to oxidative stress, aconitase, a key enzyme in the Krebs cycle, has demonstrated altered activity in CF models, including a notable loss of mitochondrial aconitase activity in FABP *Cftr*-KO mice and a decrease in CFTR-deficient lung cells, highlighting its vulnerability to oxidative damage in CF (Velsor et al., 2006). Concurrently, fumarase, another Krebs cycle enzyme, showed comparable or unvaried activity in these models, suggesting selective impact on aconitase (Velsor et al., 2006). Furthermore, γ-glutamyltransferase (γ-GT), involved in GSH metabolism, was not significantly altered in the lung tissue of *Cftr*-KO mice (Velsor et al., 2001). In immune cells, specifically activated neutrophils from CF patients, increased peroxidase activity, likely reflecting elevated MPO activity, is observed in response to stimuli such as *P. aeruginosa* (Yoo et al., 2014), contributing to the heightened oxidative burst.

## 8 Discussion

The comprehensive examination of analytical methodologies presented in this review reveals the extensive array of detection strategies available for oxidative stress assessment, including targeted molecular probes, mass spectrometry platforms, fluorescence and luminescence-based systems, ELISA protocols, and enzymology. Each analytical approach contributes distinct insights into the complex redox environment characteristic of CF pathophysiology, offering varying degrees of specificity and sensitivity in capturing oxidative-antioxidative imbalances. A fundamental understanding of the mechanistic principles underlying each analytical methodology and their specific applications remains essential for appropriate technique selection and data interpretation. While certain approaches excel in providing comprehensive assessments of total antioxidant capacity or global oxidative damage markers, others demonstrate superior capability in characterizing individual reactive oxygen species, specific antioxidant concentrations, or enzymatic antioxidant activities. The distinction between techniques offering broad-spectrum analysis versus those providing molecular-level specificity represents a critical consideration in experimental design. The strategic selection of appropriate biological specimens, whether peripheral blood components, induced sputum, BALF, exhaled breath condensate, or cultured cellular systems, significantly influences the clinical relevance and interpretability of oxidative stress measurements. Each sample type presents unique advantages and limitations in representing systemic versus localized oxidative processes, with implications for understanding disease-specific pathophysiology. Importantly, the intricate and dynamic nature of oxidative stress in CF cannot be fully characterized through any single analytical technique. Our analysis strongly supports the implementation of integrated multiparametric strategies, wherein multiple complementary methodologies are systematically combined to provide more comprehensive and robust assessments. This synergistic approach enables the generation of complementary datasets that collectively offer enhanced understanding of redox dysregulation complexities, revealing biochemical interactions that may remain undetected through isolated measurements. The combination of global oxidative damage assessments with targeted analyses of specific reactive species and individual antioxidant pathway components exemplifies this integrative approach. Such comprehensive profiling strategies are particularly valuable for elucidating the multifaceted relationships between oxidative stress mechanisms and CF progression, facilitating the development of more effective and targeted therapeutic interventions.

To contextualize the importance of these methodologies, it is useful to summarize the current understanding of oxidative stress in CF and the impact of recent therapeutic breakthroughs. It is well-established that oxidative stress in CF is a chronic and pivotal feature of the disease, driven by both intrinsic CFTR dysfunction and the relentless inflammatory response, particularly from neutrophils (Recchiuti et al., 2019; Moliteo et al., 2022). The advent of highly effective CFTR modulator therapy, especially the elexacaftor/tezacaftor/ivacaftor (ETI) combination, has revolutionized CF care by targeting the root cause of the disease. Recent studies have consistently shown that long-term ETI treatment leads to a significant reduction in systemic markers of lipid and protein damage (Liessi et al., 2023), coupled with an improvement in the antioxidant capacity, including the restoration of glutathione levels (Borcherding et al., 2019). This improvement in redox balance is thought to be mediated by both direct effects on CFTR function and a profound reduction in pro-inflammatory stimuli and neutrophil activity (Schnell et al., 2023; Jarosz-Griffiths et al., 2024). This highlights that while direct antioxidant therapies remain of interest, correcting the primary CFTR defect is a powerful strategy to re-establish redox homeostasis in patients with CF.

Looking forward, the continued advancement of analytical technologies, including real-time monitoring systems, nanotechnology-based platforms, and artificial intelligence-enhanced data interpretation, promises to further enhance our understanding of oxidative stress in CF. These developments hold significant potential for addressing the complex interplay between chronic infection, persistent inflammation, and progressive tissue damage that characterizes this condition, ultimately contributing to improved patient outcomes through personalized antioxidant therapeutic strategies.
