# Supplementary material for: Mapping the oxidative landscape in cystic fibrosis: methodological frontiers and application
Source: Front Pharmacol. 2025 Jul 16;16:1632924. doi: 10.3389/fphar.2025.1632924 (PMC12307221; doi:10.3389/fphar.2025.1632924)
Supplement: Supplementary file 1 [file Table1.docx]

Supplementary Material

**Table S1: Schematic overview of the main features regarding *in vivo* models of CF.** The table recapitulates the advantages and disadvantages of the animal models developed to study CF and gives an overview of their primary applications.

| Animal Model | Key Advantages | Key Disadvantages | Primary Applications in CF Research |
| --- | --- | --- | --- |
| Mouse | Low cost, short lifespan, advanced genetic tools, easy handling. | Lack of spontaneous human-like lung disease; severe intestinal pathology (often lethal). | Studying intestinal pathophysiology, screening of systemic drugs, basic studies of CFTR mechanisms. |
| Rat | Larger size than mice (facilitates surgical procedures/sampling); physiology is in some aspects closer to humans. | Like mice, fails to develop a fully representative lung pathology. Fewer genetic tools available. | Physiology studies where mouse size is a limitation. |
| Ferret | Develops human-like lung, pancreatic, and intestinal disease from birth. Excellent lung anatomy. | High cost, complex husbandry, longer lifespan, fewer specific reagents available. | Studying early pathogenesis of lung disease, bacterial infections, and inhaled therapies. |
| Pig | Considered the *gold standard*. Anatomy, physiology, and multi-organ pathology (lungs, pancreas, liver) are nearly identical to humans. | Very high cost, requires large-animal facilities, long lifespan, complex ethical considerations. | Translational studies, testing advanced therapies (e.g., gene therapy), studying disease from birth. |
| Zebrafish & *Drosophila* | Rapid life cycle, low cost, ideal for large-scale (high-throughput) screening, transparent embryos (zebrafish). | Physiology is very distant from mammals (no lungs). Cannot model complex organ pathology. | High-throughput screening of compounds (CFTR correctors/potentiators), basic gene function studies. |

**Table S2: Comprehensive overview of analytical methodologies for assessing oxidative stress markers and antioxidant capacity.** The table summarizes various methods by detailing their principle, typical sample types, measurement technique employed, key experimental parameters, and crucial considerations for application.

| Method | Principle | Sample Type | Measurement Technique | Key Details (e.g., Wavelengths, Products) | Considerations |
| --- | --- | --- | --- | --- | --- |
| *Superoxide Anion* | | | | | |
| Ferricytochrome c Reduction Assay | Superoxide reduces ferricytochrome c. | Extracellular | Spectrophotometry | Absorbance monitored at 550 nm (maximum for reduced ferricytochrome c). Superoxide contribution determined by difference in absorbance with/without SOD. | Other molecules (GSH, ascorbate) can also reduce ferricytochrome c. Catalase can be included to eliminate H_2_O_2_-mediated reactions. Recommended to measure samples in parallel. |
| HE/DHE | Intracellular superoxide oxidizes HE to fluorescent 2-hydroxyethidium (2-E+OH). | Intracellular | Fluorescence Microscopy, Flow Cytometry | Excitation λ≈500 nm, Emission λ≈600 nm. Increase in fluorescence proportional to superoxide. | Other ROS or RNS do not form the same fluorescent product. Specificity can be confirmed by competition with SOD. |
| MitoSOX Red | Derivative of HE targeted to mitochondria. Oxidized by mitochondrial superoxide to a fluorescent product. | Mitochondrial (in live cells) | Fluorescence Microscopy, Flow Cytometry | Similar principle to HE, targeted to mitochondria. | Designed specifically for mitochondrial superoxide detection. |
| Adrenochrome | Superoxide oxidizes epinephrine, leading to the formation of colored adrenochrome. | Not specifically limited, implies reaction mixtures. | Spectrophotometry | Change of absorbance at 485 nm monitored. | Epinephrine and adrenochrome can be metabolized by NADPH-CYP reductase, making the assay inappropriate for studying superoxide generation during CYP-mediated metabolism of xenobiotics. |
| *Hydrogen Peroxide* | | | | | |
| DCFH-DA | Membrane-permeable DCFH-DA is deacetylated intracellularly to DCFH, which is then oxidized by intracellular H_2_O_2_ to fluorescent DCF. | Intracellular | Fluorescence Microscopy, Flow Cytometry, Spectrophotometry | Excitation λ=488 nm, Emission λ=530 nm for DCF. | DCFH can potentially react with other ROS/RNS, though primarily used for H_2_O_2_. |
| Peroxidase-mediated Assays | H_2_O_2_ oxidizes susceptible probes in the presence of a peroxidase (e.g., HRP), resulting in a change in fluorescence or absorbance. | Extracellular | Spectrophotometry, Fluorometry | Scopoletin: Fluorescence bleached upon oxidation. HVA: Fluorescent dimer formed (Excitation λ=321 nm, Emission λ=421 nm). Phenol Red: Absorbance at 610 nm. ADHP (Amplex Red^®^): Oxidized to fluorescent resorufin (Excitation λ=561 nm, Emission λ=585 nm). pHPA: Fluorescent dimer formed (Excitation λ=320 nm, Emission λ=400 nm). | ADHP oxidation by other ROS can occur at lower yield than H_2_O_2_. ADHP signal is inhibitable by catalase, confirming H_2_O_2_ specificity. ADHP offers low background and high sensitivity. |
| *Lipid Hydroperoxides* | | | | | |
| TBARS | Malondialdehyde (MDA), a lipid peroxidation product, reacts with thiobarbituric acid (TBA) under acidic conditions to form colored TBARS adducts. | Implies biological samples containing lipids. | Spectrophotometry, Fluorometry | Absorbance at 532 nm or Fluorescence (Excitation λ=370 nm, Emission λ=420 nm). | Not all lipid peroxidation forms MDA. Other molecules react with TBA. MDA is not exclusively from lipid peroxidation. Experimental conditions can cause artefactual product formation. Recommended to associate with other methods or use improved methods. |
| FOX1 Assay | Lipid hydroperoxides oxidize ferrous (Fe^2+^) to ferric (Fe^3+^) ions. Ferric ions chelate with xylenol orange to form a colored complex. | Aqueous phase samples with low lipid hydroperoxide levels. | Spectrophotometry | Absorbance detected at 560 nm. | FOX1 is described as more sensitive than FOX2 (for lipid phase). |
| PnAc | Naturally fluorescent fatty acid that localizes to membranes. Fluorescence decreases upon oxidative stimuli (lipid peroxidation). | Membranes | Spectrofluorometer, Flow Cytometer | Natural fluorescence at λ≈420 nm when excited at λ≈320 nm. Decrease in fluorescence indicates lipid peroxidation. | Fluorescence is decreased by oxidative stimuli. |
| BODIPY™ 581/591 C11 | Lipid peroxidation sensor that localizes to membranes. Oxidation causes a shift in fluorescence emission. | Membranes | Spectrofluorometer, Flow Cytometer | Reduced form emission peak at ≈590 nm. Oxidized form emission peak shifts to ≈510 nm. Allows ratiometric analysis. | Fluorescence emission shift upon oxidation. |
| MitoPerOx | Derivative of BODIPY™ 581/591 C11 targeted to the inner mitochondrial membrane by a triphenylphosphonium cation. Detects mitochondrial lipid hydroperoxides. | Inner mitochondrial membrane | Spectrofluorometer, Flow Cytometer | Oxidation induces a shift in fluorescence emission peak from ≈590 nm to ≈520 nm. | Specificity for the inner mitochondrial membrane. Uptake driven by mitochondrial membrane potential. |
| *Mitochondrial functionality* | | | | | |
| Seahorse XF | Real-time, label-free, non-destructive measurement of Oxygen Consumption Rate (OCR) and Extracellular Acidification Rate (ECAR). | Adherent or suspended cell cultures | Extracellular Flux Analysis | Measures OCR (mitochondrial oxidative phosphorylation) and ECAR (glycolysis). Allows assessment of basal/maximal respiration, ATP-linked respiration, glycolytic reserve. | Focuses on cellular bioenergetics (ATP generation pathways) rather than direct ROS measurement, though related to oxidative phosphorylation. |
| *DNA damage* | | | | | |
| Separative Methods  (HPLC, GC-MS) | Separation of guanine oxidation products 8-OHdG and 8-oxoGua. | Biological samples containing cells | HPLC, Gas Chromatography (GC) | Often coupled with mass spectrometer detectors with selective ion monitoring for increased sensitivity. | Chromatographic techniques are considered more reliable than ELISA. |
| ELISA | Immunoassay-based detection of guanine oxidation products 8-OHdG and 8-oxoGua. | Biological samples containing cells | ELISA | Based on the recognition antigen-antibody. Colorimetric assay. | Lack of accuracy and precision. A highly specific antibody is required. DNA isolation step can induce sample oxidation. Availability of commercial kit. |
| Alkaline unwinding | dsDNA unwinds in an alkaline solution and each strand break is an initial point of unwinding. | Biological samples containing cells | Chromatography,  Spectrofluorometer | After unwinding, dsDNA residual is inversely proportional to strand breaks amount. Fluorescence intensity is higher for dsDNA than ssDNA. Excitation and emission wavelength depend on the DNA-binding dye of choice. | Differently from Comet assay, isolation of intact cells is not required. Cell lysis could produce artefactual strand breaks. High number of cells is required. |
| Comet assay | Strand breaks induce DNA supercoiling relaxation and allow DNA migration. | Biological samples containing cells | Gel Electrophoresis, Fluorescence Microscopy | The % of DNA in the tail is indicative of DNA strand breaks.  Excitation and emission wavelength depend on the DNA-binding dye of choice. | High sensitivity. Single cell preparation procedures could alter DNA damage amount. Small cell samples could introduce bias. |
| *Nonenzymatic Antioxidants (GSH)* | | | | | |
| GSH-GSSG recycling assay | Based on GR recycling of GSSG to GSH. GSH reacts with DTNB to form yellow TNB, measured by absorbance. GR regenerates GSH from GS-TNB, amplifying signal. | Intracellular (implies biological samples containing cells). | Spectrophotometry | Uses Ellman’s reagent (DTNB) and Glutathione Reductase (GR). Measures absorbance at 412 nm due to TNB formation. Measures total GSH ([GSH] + 2 x [GSSG]). | Requires masking of reduced GSH. GSSG quantification was considered difficult due to low GSH/GSSG ratio and favored GSH oxidation. |
| Separative Methods (HPLC, GC, CE) | Separation of GSH/GSSG from complex biological matrices before detection. | Biological samples containing cells | HPLC, Gas Chromatography (GC), Capillary Electrophoresis (CE) | Often coupled with electrochemical or mass spectrometer detectors for increased sensitivity. | Requires sample preparation for separation. |
| *Nonenzymatic Antioxidants (Vitamins)* | | | | | |
| LC-MS/MS | Chromatographic separation of vitamins and metabolites followed by sensitive and specific detection by mass spectrometry. | Serum or plasma | LC-MS/MS | Requires sample preparation (protein precipitation, liquid-liquid extraction). Allows simultaneous quantification of multiple vitamins. | Regarded as a gold standard due to sensitivity and specificity. Particularly advantageous for limited sample volumes (e.g., pediatric patients). |
| HPLC | Chromatographic separation of specific vitamins followed by detection. | Serum or plasma | HPLC with UV or fluorescence detectors | Utilizes similar extraction procedures as LC-MS/MS. | Well-established and reliable for quantifying specific vitamins. |
| ELISA | Immunoassay-based detection of specific vitamins. | Serum or plasma | ELISA | Based on the recognition antigen-antibody. Colorimetric assay. | More commonly applied for specific vitamins (e.g., Vitamin D). |
| *Total Antioxidant Capacity (TAC)* | | | | | |
| ABTS | Radical quenching diminishes light absorption. | Biological samples | Spectrophotometry | Measures absorbance of the ABTS•^+^ radical cations at 734 nm. | Highly soluble in both organic and aqueous solvents. Detects both SET and HAT.  Often Trolox is used as reference standard. |
| FRAP | Reduction of Fe³⁺-TPTZ to Fe^2^⁺-TPTZ with generation of a chromophore. | Serum or plasma | Spectrophotometry | Measures absorbance of the chromophore at 593 nm. Requires an acidic environment (pH 3.6). | Potentially underestimate HAT. Often ferrous ion equivalents or Trolox are used as reference standard. |
| DPPH | Radical quenching diminishes light absorption. | Biological samples | Spectrophotometry | Measures absorbance of the DPPH• radical cations at 517 nm. | Detects both SET and HAT. Often ascorbic acid or Trolox are used as reference standard. |
| BAP | Reduction of ferric-chromogen complexes. | Biological samples | Spectrophotometry | Measures absorbance of ferrous products at 505-520 nm (according to the commercial kit). | High throughput. Often ferrous ion-based standards are used as a reference. |
| TRAP | Neutralization of  R-OO•. | Biological samples (in particular plasma) | Spectrofluorometer, Luminometer | Fluorescence signal deterioration or light emission decay (according to the probe of choice) are consequences of oxidation. | More complex and time consuming than ABTS, FRAP, DPPH and BAP. Trolox can be used as a reference standard. |

**Table S3:** **Overview of proteomics methodologies for oxidative stress characterization in CF.** The table summarizes various techniques, detailing their principles, specific applications to oxidative stress, key strengths, and limitations.

| Methodology | Principle | Specific Application to Oxidative Stress | Key Strengths | Key Limitations |
| --- | --- | --- | --- | --- |
| *Gel-Based Proteomics* | | | | |
| 2D-PAGE | Separation of complex protein mixtures. Two Steps: isoelectric point, then molecular weight. | Applied in CF BALF proteome analysis, differential abundance of low molecular weight proteins and degradation of critical proteins. | Protein separation; capability to resolve thousands of protein spots; useful for revealing significant global protein alterations. | Labor-intensive; restricted dynamic range; suboptimal resolution of proteins with extreme molecular weights or high hydrophobicity. |
| 2D-DIGE | Quantitative comparative analysis of multiple protein samples on a single gel by pre-labeling with distinct fluorescent chromophores. | Applied in CF serum proteomics to identify differentially abundant proteins, which include oxidative stress markers or proteins altered by it. | Facilitates quantitative comparative analysis of multiple samples concurrently. | Same limitations as 2D-PAGE. |
| *Mass Spectrometry (MS)-Based Proteomics* | | | | |
| Shotgun (LC-MS/MS) | Enzymatic digestion of proteins into peptides, LC separation, followed by MS analysis. | Widely applied to the analysis of diverse biological samples relevant to CF research (BALF, serum). | Unparalleled sensitivity, specificity, and throughput for protein identification and quantification. | Limitations compared to specific quantitative/targeted methods for certain aspects of oxidative stress. |
| MudPIT | Incorporates two or more orthogonal chromatographic separation stages preceding MS analysis. | Effectively utilized to identify hundreds of differentially expressed proteins in CF cellular models, elucidating proteins implicated in CF pathogenesis and CFTR proteostasis regulation, aspects influenced by oxidative stress. | Significantly enhanced proteome coverage compared to single-dimension LC-MS/MS. | Similar to general shotgun proteomics. |
| *Quantitative MS-Based Proteomics Strategies* | | | | |
| LFQ | Comparison of protein abundance based on MS signal intensity or spectral counts, without isotopic labels. | Extensive application in CF research: CF cellular models, metaproteomic analyses of BALF, and plasma proteomics in IPF. Useful for monitoring global changes induced by oxidative stress. | Simplified sample preparation protocols; adaptability to virtually any sample type; frequently yields comprehensive proteome coverage. | Quantitative accuracy and reproducibility may be comparatively lower than label-based techniques in some instances, particularly for detecting subtle modulations in protein expression. |
| SILAC | Metabolic labeling: cells cultured in media with “light” or “heavy” (stable isotope-enriched, e.g., 13C-lysine, 15N-arginine) essential amino acids. Relative protein abundance is determined by the ratio of heavy to light peptide signals in MS. | Used to compare the proteome of apical secretions from CF and non-CF human bronchial epithelial cells, identifying CFTR-dependent changes. Ideal for dissecting fundamental mechanisms of oxidative stress in controlled cell culture models. | High accuracy and precision in cell culture experiments due to early sample mixing, minimizing downstream processing variability. | Applicability mainly to metabolically active, culturable cells; less suitable for direct analysis of patient tissues or body fluids. |
| iTRAQ, TMT | Chemical labeling of peptides with isobaric tags. Fragmentation in MS/MS releases unique “reporter ions” whose intensity reflects relative peptide/protein abundance. | Well-suited for analyzing complex biological samples (tissues, body fluids) and studying PTMs and oxidative stress in CF. | Increased throughput and statistical power by concurrent analysis of multiple conditions. | “Ratio compression” issue: co-isolation/co-fragmentation of interfering peptides can lead to an underestimation of true fold changes. Chemical labeling steps add cost and complexity to the workflow. |
| *Targeted MS-Based Proteomics for Biomarker Validation* | | | | |
| MRM/SRM | Hypothesis-driven approach for precise and sensitive quantification of a pre-selected list of target proteins/peptides. The MS is programmed to selectively monitor specific precursor ion-to-fragment ion transitions unique to target peptides. | Absolute quantification of the CFTR protein. Validation of differential expression of candidate protein biomarkers in CF serum. Critical for validating oxidative stress biomarkers in larger patient cohorts. | Precise and sensitive quantification of pre-selected targets; high selectivity; robust, reproducible, and high-throughput for biomarker validation. | Hypothesis-driven, so it only measures pre-selected targets; not for discovery of new biomarkers. |
| *Redox Proteomics (MS-Based)* | | | | |
| Carbonylation | Identifies proteins with carbonyl groups (aldehydes or ketones), a marker of severe oxidative protein damage. Derivatization with DNPH or tagged hydrazide reagents for enrichment and LC-MS/MS identification of sites. | CF patients exhibit elevated systemic levels of protein carbonyls. The “carbonylome” is still not provided in CF. | Directly identifies and quantifies a key irreversible oxidative PTM; can pinpoint specific sites of carbonylation; | General PTM analysis complexities. |
| S-Nitrosylation | Identifies S-nitrosylation (SNO). Reversible PTM. Proteomic identification often relies on “biotin-switch” type assays. | Excessive NO production/nitrosative stress in CF airways can dysregulate S-nitrosylation patterns, contribute to pathology. | Biotin-switch method allow for enrichment and MS identification. | SNOs can be labile, making detection challenging; complexity of the biotin-switch assay and potential for non-specific labeling. |
| S-Glutathionylation | Identifies S-glutathionylation. Reversible PTM. Trap glutathionylated peptides; isotopically labeled clickable glutathione derivatives for enrichment and MS. | CFTR dysfunction impairs GSH transport/homeostasis, likely perturbing S-glutathionylation. Profiling the S-glutathionylome elucidate how signaling and protective mechanisms are perturbed. | Identifies a key protective and regulatory redox PTM; advanced techniques allow for site identification and quantification; good for understanding response to disrupted GSH homeostasis in CF. | Similar to S-nitrosylation, lability of the modification can be a challenge; complexity of enrichment and identification strategies. |
| *Advanced MS-Based Techniques for Studying Protein Oxidation* | | | | |
| FPOP | MS-based footprinting. Utilizes hydroxyl radicals generated by laser-induced photolysis to map solvent-accessible protein surfaces. Sites and extent of modification are identified by MS analysis of the oxidized protein/peptides. | Highly promising for CF oxidative stress. FPOP mimics and maps sites susceptible to hydroxyl radical attack. Specific applications in CF oxidative stress still emerging. | Maps solvent-accessible surfaces with residue-level resolution; well-suited for dynamic processes due to rapid labeling; can study proteins in their native environment (IC-FPOMP); provides insights into how modifications occur and their structural impact. | Requires specialized equipment (laser); data analysis can be complex; potential for non-specific modifications or radical-induced damage beyond simple footprinting. |

**Table S4: Summary of lipidomic approaches applied in CF contexts.** The table describes various mass spectrometry-based analytical platforms and a lipid extraction technique, highlighting their purpose and key features or considerations pertinent to CF research.

| Approach/Technique | Description & Purpose | Key Features/Considerations in CF Research |
| --- | --- | --- |
| Mass Spectrometry-based Lipidomics | Overall approach for studying lipid dysregulation. | Indispensable for elucidating the complex relationship between lipid dysregulation and oxidative stress in CF. Optimized sample preparation is key. |
| Liquid Chromatography-Mass Spectrometry (LC-MS) | Separates lipids by physicochemical properties before MS detection. | Improves detection of structurally similar lipids (isobaric/isomeric species). Uses High-Resolution Mass Spectrometry (HRMS - Orbitrap, TOF) for accurate mass measurements and confident identification. |
| Matrix-Assisted Laser Desorption/Ionization Time-of-Flight MS (MALDI-TOF-MS) | Mass spectrometry platform. | Frequently coupled with Thin Layer Chromatography (TLC-MALDI). |
| Direct Infusion MS (Shotgun Lipidomics) | Direct introduction of lipid extract into MS. | High-throughput for lipid class profiling; requires very high mass resolution and/or MS/MS for confident differentiation of isobaric species. |
| *Extraction Methods* | | |
| Bligh and Dyer Method | Utilizes organic solvent systems (e.g., chloroform/methanol). | Common approach for effective solubilization and recovery of diverse lipids from biological samples. Tailored to the specific matrix. |

**Table S5: Overview of common assay methodologies for the antioxidant enzymes SOD, CAT, and GPx.** This table outlines the assay principle, typically utilized biological samples, common normalization strategies, and key practical considerations for each discussed method.

| Assay Principle | Common Biological Samples | Typical Normalization | Key Considerations |
| --- | --- | --- | --- |
| *Superoxide Dismutase (SOD)* | | | |
| Spectrophotometric (e.g., Xanthine Oxidase/NBT or WST-1, Cytochrome c reduction) | Erythrocytes, Plasma, Serum, Sputum, BALF, Tissue/Cell Lysates | Hb, Protein, Volume | Measures total SOD activity; can be adapted for high throughput. |
| In-Gel Zymography | Tissue/Cell Lysates, Erythrocytes, Sputum | (Semi-quantitative) | Allows differentiation of isoenzymes (Cu/Zn-SOD, Mn-SOD, EC-SOD). |
| *Catalase (CAT)* | | | |
| Spectrophotometric (Aebi method - H_2_O_2_ decay at 240 nm) | Erythrocytes, Tissue/Cell Lysates, Sputum | Hb, Protein, Volume | Direct measurement of H_2_O_2_ decomposition; widely used. |
| Spectrophotometric (Chromogenic for residual H_2_O_2_) | Sputum, Tissue/Cell Lysates | Protein, Volume | Indirect measurement; e.g., pHPA/horseradish peroxidase or hydroquinone-based. |
| In-Gel Zymography | Tissue/Cell Lysates, Erythrocytes | (Semi-quantitative) | Visualizes catalase activity bands. |
| Radioimmunoassay (RIA) | Sputum | Volume | Measures enzyme protein concentration, not activity. |
| *Glutathione Peroxidase (GPx)* | | | |
| Spectrophotometric (Paglia & Valentine - coupled NADPH oxidation) | Erythrocytes, Plasma, Serum, Sputum, Tissue/Cell Lysates | Hb, Protein, Volume | Indirect; monitors NADPH decrease at 340 nm; most common method. |
| Direct GSH Consumption Assays | Erythrocytes | Hb, Protein | Measures decrease in GSH over time. |
| In-Gel Zymography | Tissue/Cell Lysates | (Semi-quantitative) | Visualizes GPx activity bands. |
